# Using Cortical Neuron Markers to Target Cells in the Dorsal Cochlear Nucleus

**DOI:** 10.1523/ENEURO.0413-20.2020

**Published:** 2021-02-25

**Authors:** Thawann Malfatti, Barbara Ciralli, Markus M. Hilscher, Steven J. Edwards, Klas Kullander, Richardson N. Leao, Katarina E. Leao

**Affiliations:** 1 Hearing and Neuronal activity Lab, Brain Institute, Federal University of Rio Grande do Norte, Natal, Brazil 59056-450; 2 Institute for Analysis and Scientific Computing, Vienna University of Technology, Vienna, Austria 1040; 3 Unit of Developmental Genetics, Department of Neuroscience, Uppsala University, Uppsala, 75237 Sweden

**Keywords:** CaMKIIa, Chrna2, dorsal cochlear nucleus, extracellular recording, optogenetics, ventral cochlear nucleus

## Abstract

The dorsal cochlear nucleus (DCN) is a region of particular interest for auditory and tinnitus research. However, lack of useful genetic markers for *in vivo* manipulations hinders elucidation of the DCN contribution to tinnitus pathophysiology. This work assesses whether adeno-associated viral vectors (AAV) containing the calcium/calmodulin-dependent protein kinase 2α (CaMKIIα) promoter and a mouse line of nicotinic acetylcholine receptor α2 subunit (Chrna2)-Cre can target specific DCN populations. We found that CaMKIIα cannot be used to target excitatory fusiform DCN neurons as labeled cells showed diverse morphology indicating they belong to different classes of DCN neurons. Light stimulation after driving Channelrhodopsin2 (ChR2) by the CaMKIIα promoter generated spikes in some units but firing rate decreased when light stimulation coincided with sound. Expression and activation of CaMKIIα-eArchaerhodopsin3.0 in the DCN produced inhibition in some units but sound-driven spikes were delayed by concomitant light stimulation. We explored the existence of Cre+ cells in the DCN of Chrna2-Cre mice by hydrogel embedding technique (CLARITY). There were almost no Cre+ cell bodies in the DCN; however, we identified profuse projections arising from the ventral cochlear nucleus (VCN). Anterograde labeling in the VCN revealed projections to the ipsilateral superior olive and contralateral medial nucleus of the trapezoid body (MNTB; bushy cells), and a second bundle terminating in the DCN, suggesting the latter to be excitatory Chrna2+ T-stellate cells. Exciting Chrna2+ cells increased DCN firing. This work shows that cortical molecular tools may be useful for manipulating the DCN especially for tinnitus studies.

## Visual Abstract

**Figure:**
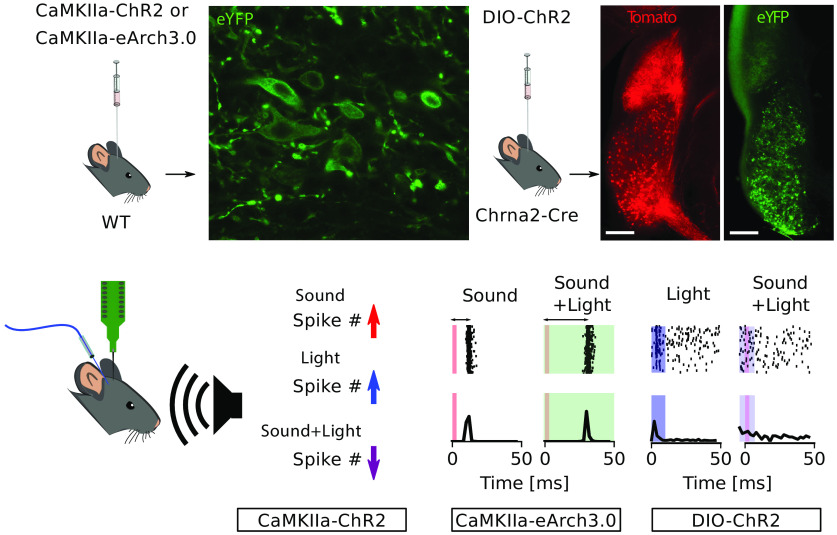

## Significance Statement

Cortical neuron markers could be used to label subcortical regions such as the dorsal cochlear nucleus (DCN) that integrates sound and somatosensory input. Here, we examine whether excitatory (CaMKIIα) or inhibitory [nicotinic acetylcholine receptor α2 subunit (Chrna2)] neuron markers used in neo/paleo-cortex studies label unique DCN populations. We found CaMKIIα to be expressed by different DCN neuron populations (affecting sound sensitive and nonsensitive neurons). The Chrna2 promoter was specifically expressed in excitatory cells of the ventral cochlear nucleus (VCN) and could drive indirect activity in the DCN. This study highlights novel ways of regulating DCN neuron activity, which can provide new means for treatment of bothersome tinnitus.

## Introduction

The dorsal cochlear nucleus (DCN) of the auditory brainstem is the first integrator of auditory and multisensory signals and has been pointed as a key structure in tinnitus physiopathology (Kaltenbach et al., 2005; Tzounopoulos, 2008; Baizer et al., 2012). Cells in the DCN receive direct or indirect [e.g., relayed by the ventral cochlear nucleus (VCN)] sound input onto different cell populations in a layer arrangement. The most cell-populated DCN field is the fusiform cell layer formed by excitatory fusiform cells intercalated with interneurons (Oertel and Young, 2004). An interesting aspect of the DCN is its architectural similarity to the cerebellum (Devor, 2000) that is thought to be responsible for integrative processing (e.g., sound/somatosensory; Oertel and Young, 2004).

Abnormal sensory integration in the DCN is clinically relevant because of the prevalence of temporomandibular tinnitus (Levine, 1999; Grossan and Peterson, 2017). Other forms of mechanical tinnitus are also attributed to aberrant activity in the DCN (Han et al., 2009). Also, a large number of studies have shown altered synaptic and intrinsic cellular properties within the DCN circuit relating to noise-induced tinnitus (for review, see Shore et al., 2016) yet tinnitus treatments to date do not specifically target this region. The VCN can also contribute to noise-induced tinnitus (Kraus et al., 2011; Coomber et al., 2015). Aberrant activity in any cochlear nucleus subregions can trigger upstream changes as cochlear nucleus neurons relay auditory signals to higher areas of the auditory pathway (Kraus et al., 2011; Coomber et al., 2015). Abnormal activity from auditory cortex and inferior colliculus can also produce downstream alterations in the DCN as its cells receive feedback through descending auditory fibers (Winer and Prieto, 2001; Milinkeviciute et al., 2017). Despite its physiological importance and its well accepted role in tinnitus, the contribution of specific DCN populations to hearing and tinnitus pathophysiology are largely unknown.

Because of its variety of cell types and its cerebellum like structure, DCN circuit studies could benefit from identifying key neuronal markers (Hilscher et al., 2017). The Calcium/calmodulin-dependent protein kinase 2α (CaMKIIα) promoter is widely used for targeting cortical pyramidal cells. Immunohistochemical data from rats has shown CaMKIIα expression in the DCN molecular and fusiform cell layers (Ochiishi et al., 1998). In mice CaMKIIα RNA is widely distributed in the fusiform layer (Lein et al., 2007). Hence, viral vectors to express reporter or optogenetic proteins by the CaMKIIα promoter may be applied to DCN manipulation. Cortical interneuron markers could also be used to tag DCN cells. However, for two commonly used markers, both parvalbumin and somatostatin expression can be quite promiscuous in the neocortex/hippocampus region (Kawaguchi and Kondo, 2002; Mikulovic et al., 2015) and in the DCN, parvalbumin is distributed across layers without apparent population specificity and somatostatin shows weak expression (Lein et al., 2007). Nonetheless, recently, the nicotinic acetylcholine receptor α2 subunit (Chrna2) has been described as a marker for highly specific interneuron populations (L5 Martinotti cells in the neocortex or CA1 oriens-lacunosum moleculare cells in the ventral hippocampus; Leão et al., 2012; Hilscher et al., 2017). Cre+ cells in Chrna2-cre mice seem also to belong to single populations in several subcortical nuclei (Mikulovic et al., 2018; Siwani et al., 2018). Whether Chrna2 is expressed in the DCN is not described.

Here, we test whether adeno-associated viral vectors (AAV) with the CaMKIIα promoter can be used for manipulating DCN circuits *in vivo*. AAV encoded optogenetic protein expression and light stimulation paired with brief sound presentation was used to functionally identify cells and assess the effect of optical depolarization/hyperpolarization in input/output functions in CaMKIIα+ neurons in combination with brief sound stimulation. Lastly, we examined how activation of Chrna2+ cells innervating the DCN modulate DCN unit activity.

## Materials and Methods

### Mice

Male C57Bl/6J mice, Chrna2-cre or Chrna2-cre mice crossed with the reporter line Ai14 tdTomato (Chrna2-Cre/R26^tom^) mice age postnatal day (P)21–P75 (*n* = 20) were used in this study. All animal procedures were approved by the Federal University of Rio Grande do Norte Ethical Committee in Use of Animals (CEUA - protocol number 051/2015) and followed the guidelines for care and usage of laboratory animals of the Federal University of Rio Grande do Norte.

### Virus injection of optogenetic constructs

Approximately four weeks before experiments using optogenetic stimulation, mice were injected with viral constructs of different opsins coupled to either channelrhodopsin2 (ChR2) or Archaerhodopsin3.0 (eArch3.0). ChR2 is a light activated cation permeable channel (Boyden et al., 2005) for membrane depolarization, while eArch3.0 is a green light activated outward proton (H^+^) pump (Chow et al., 2010) for membrane hyperpolarization. ChR2 constructs used were: rAAV5/CaMKIIα-hChR2(H134R)-EYFP (Vector core, at a concentration of 4 × 10^12^ virus molecules – vm/ml) and a cre-dependent (double-floxed inverted open reading frame; DIO) ChR2 construct, AAV2/9.EF1a.DIO.hChR2(H134)-eYFP-WPRE-hGH (Vector core, at 1 × 10^13^ vm/ml). The eArch3.0 used was rAAV5/CamK2a-eArch3.0-eYFP (Vector core, at 2.5 × 10^12^ vm/ml). In detail, mice were anesthetized with ketamine-xylazine at 90/6 mg/kg intraperitoneal. If required, additional ketamine was re-administered (as half the dose of the previous injection, i.e., 45 and 22.5 mg/kg) during surgery. The mouse was mounted into a stereotaxic device while resting on a heating block at 37°C. Eye gel (dexpanthenol) was applied to avoid drying of eyes during surgery. The head was wiped with polyvidone-iodine (10%) to avoid infections. The skin was anesthetized with lidocaine hydrochloride 3% before a straight incision was made. After the incision, hydrogen peroxide 3% was applied onto the exposed skull to remove the connective tissue and to visualize sutures.

The DCN coordinates were taken from Franklin and Paxinos (2008). Specifically, we used −6.1 mm anteroposterior (AP), −2.3 mm mediolateral (ML), and −4.3 and −3.8 mm dorsoventral (DV; two steps). For each animal, those coordinates were corrected by multiplying by the normalized bregma-lambda distance (mouse’s bregma-lambda in mm divided by 4.2 – the average bregma-lambda distance from the mice used in Paxinos and Franklin’s atlas), to account for head size differences. Additionally, the vertical distance between the bregma and the point in the skull at the AP and ML coordinate was subtracted from the DV coordinate, so that the DCN can be reached using the brain surface as reference. A small mark was made at the AP and ML coordinates and a small hole was carefully drilled with a dental microdrill (Beavers Dental). Next, prealiquoted virus (20% for Cre-dependent, 30% for CaMKIIα-dependent vectors) was rapidly thawed and withdrawn into a microsyringe (10 μl Nanofil, 34-gauge removable needle), at the speed of 1.5 μl/min using a infusion pump (Chemyx NanoJet). The syringe was lowered into the DCN to the deepest DV coordinate, and 0.75 μl of virus was slowly infused at 0.15 μl/min and the needle was kept in place for 5 min to allow for full diffusion of virus, then retracted to the second, more superficial DV coordinate for a second infusion (0.75 μl) of virus and the needle kept in place for 10 min, before carefully removed. Some animals received bilateral injections. Next, the skin was sutured, lidocaine hydrochloride 3% was applied over the suture and 200 μl of saline was injected subdermal in the back for rehydration. The animal was removed from the stereotaxic frame and placed under a red heat lamp and monitored until recovering from anesthesia. Some initial experiments used fluorescent retrobeads (Green fluorescent retrobeads, Lumafluore) to establish the appropriate coordinates of injection. The benefits of initially using retrobeads, compared with viral injections of optogenetic material during optimization of experimental procedures, are (1) the fluorescent liquid can be readily seen withdrawn into the microsyringe with the naked eye (compared with a minute volumes of transparent viral solution that sometimes fails to be withdrawn because of technical issues), and (2) animals can be killed after only a few days (compared with waiting two to four weeks for viral expression) to confirm the appropriate location of fluorescent signal.

### Sound calibration and sound stimulation

As different sound devices can have inherent shifts in unit level, and thereby in the signal generation, the sound card (Sound Devices USBPre2, Thomann GmbH; 192 kHz sampling rate, 24-bit ADC) was initially calibrated using an oscilloscope. A 10 kHz sine wave of 1V amplitude was written to the card, and the sound card output amplification factor was recorded as 1 divided by the amplitude of the output signal. All sound signals were multiplied by the output amplification factor before being written to the card. We connected the sound card output to the sound card input, and a 1V 10kHz sine wave was played and recorded. The input amplification factor was measured as 1 divided by the amplitude of the recorded signal, and signals read from the board were multiplied by it before any further processing. A loudspeaker (Super tweeter ST400 trio, Selenium Pro) was calibrated using a microphone (4939-A-011, Brüel and Kjær; 1/4 free-field microphone, sensitivity of 4.23643 mV/Pa) 4.5–10 cm in front of the speaker. Sound pulses (2 s in duration) were generated at the desired frequency bands with logarithmically decreasing amplification factors (voltage output to the speaker) and simultaneously recorded using a personal computer (HP Z220; Intel Xeon 8-core 3.6 GHz, 16 GB RAM), and the power spectral density (PSD) of the recorded signal was calculated using a Hann window with no overlap. Root mean square (RMS) was calculated as

(1) $$\sqrt{\overset{n}{\underset{i=1}{\sum }}PSD_{i}\times BinSize},$$

where *PSD* is a 1*n* array and *BinSize* is the spectral resolution. The intensity in decibels sound pressure level (dBSPL) was calculated as

(2) $$20\times \log\left(\frac{\frac{RMS}{MicSens_{VPa}}}{2\times 10^{-6}}\right),$$

where MicSens_vpa_ is the microphone sensitivity in V/Pa, 0.00423643V/Pa for our microphone. All data were saved to disk and loaded to provide the correct amplification factors for the sound intensity used for sound stimulation. The frequency band generated corresponds to the frequency band of greatest power in the signal spectrum, with border frequencies strongly attenuated. Sound calibrations were routinely repeated before every beginning of an experimental group. The full sound calibration tests 300 amplification factors for the frequency band used for stimulation, providing 0.5 dBSPL precision. Sound stimulation consisted of sound pulses of Gaussian white noise filtered from 5–15 kHz, intensity of 80 dBSPL and duration of 3 ms, presented at 10 Hz (3 ms of sound pulse followed by 97 ms of silence), repeated for five blocks of 200 pulses.

### Light calibration and optogenetic stimulation

**Figure 1.**
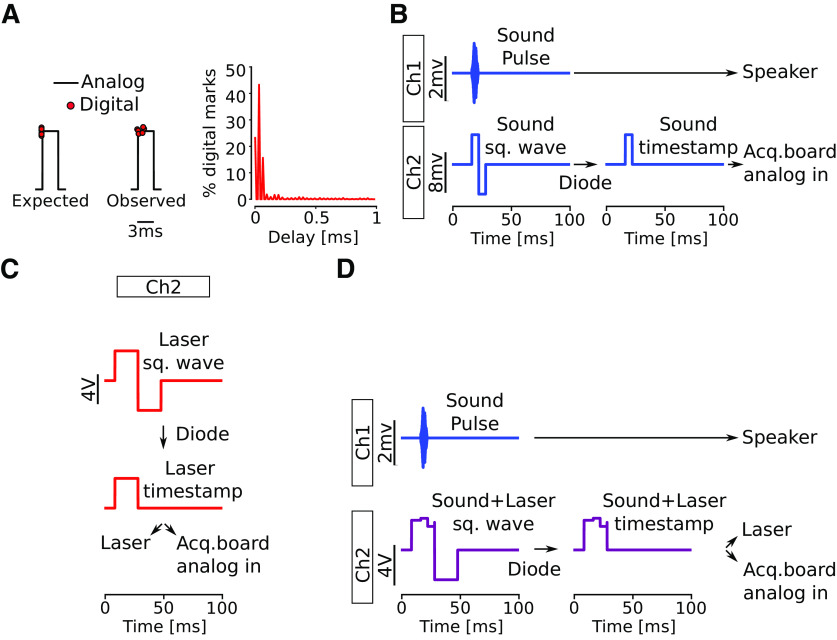
Schematics of sound and light triggers combined with digital time stamps using a two channel sound card. ***A***, Schematic showing analog pulses and a small jitter of digital detected timestamps. Right, Histogram showing percentage of recorded pulses and delay to analog edge. ***B***, Illustration of channel 1 producing a sound wave to the speaker; channel 2 producing the corresponding square wave with the positive portion with the same duration as the sound pulse (gray shading). Using a diode cuts the negative portion and can be detected as the sound timestamp. ***C***, Channel 2 writes the light square wave and the positive portion is split to provide a laser trigger and the acquisition board analog input indicates the light timestamp. ***D***, Channel 1 as in ***B***, and channel 2 illustrates the sum of light and sound square waves. As in ***C***, both timestamps can be extracted from the signal recorded from the analog input of the acquisition board.

Light intensity calibration was performed before each experiment. Optic fibers of 200 μm diameter were cleaned with lens cleaning tissue and ethanol (99.5%). The light intensity was measured and the laser lens position adjusted until light power at the tip of the fiber was 5–7 mW/mm measured by an optical power meter (Thorlabs PM20). Light stimuli triggers were generated and written to the sound card, in which the output was split and connected to the laser input and the data acquisition board (Fig. 1*C*,*D*). Light stimulus was delivered using a 473 nm laser (CNI, MBL-III-473; for ChR2) and a 532-nm laser (CNI, MGL-III-532; for eArch3.0). Laser stimuli consisted of 200 light pulses at intensity of 5–7 mW/mm with 10 ms duration, presented at 10 Hz (10 ms on and 90 ms off) 473 nm blue light for ChR2 experiments; and a pulse of a total of 20 s of 543 nm green light repeated in five blocks with 10 s interval, so that green light was continuously on during each sound stimulation block for eArch3.0 experiments. For concomitant sound and blue light stimulation, the light pulses were presented 4 ms before the sound pulses, so that these are embedded in the light pulse.

### Digital timestamps marks for sound and light stimulation

Digital timestamps markers use a combination of a sound card and an Arduino board (Arduino Due; 54 digital pins, 12 12-bit ADCs, two 12-bit DACs), taking advantage of GNU/Linux audio real-time capabilities. One output of the sound card was connected to the sound amplifier (PM8004, Marantz), and another output to an acquisition board (Open-Ephys, Open-ephys.org; v2.2, Opal Kelly XEM6010-LX150, 30 kHz sampling rate, 16 bit, eight ADCs, eight DACs) analog input with a diode as rectifier, conducting only positive voltages. In detail, the two outputs of the sound card are used for sound stimulation (channel 1) and for generating timestamp marks (channel 2). To test the temporal accuracy of the digital input of the acquisition system (Open-Ephys), we recorded 5000 square pulses delivered by both analog and digital inputs to the acquisition board. When compared with analog traces, we found 15% of the digital timestamps to be delayed by >150 μs, which is a jitter of 5% of the 3-ms pulse width (Fig. 1*A*). To avoid jitter, analog square waves to mark stimulation timestamps were used. To avoid producing capacitive-like traces when using square pulses in a sound card, we used square waves of twice the stimulus duration, containing both positive and negative portions (Fig. 1*B*). In practice, channels carrying square waves are connected to a diode before connecting to the analog input of the acquisition board or to the laser, thereby only conducting the positive values (Fig. 1*B*,*C*). The resultant square waves have the same duration as the stimulus, since only the positive half is conducted (Fig. 1*B*), thereby channel 2 was used both as timestamp marker and as a trigger for light stimulation.

For experiments using sound synchronized with light stimulation three outputs would be required (one carrying the sound signal to a speaker, one carrying the sound square waves/timestamps and the third carrying square waves for light trigger/timestamps). Here, a square wave of twice the length for light stimulation was used, so when simultaneous sound and light stimulation is required, the sound pulse is written to channel 1 while the sum of the sound and light square waves is written to channel 2 (Fig. 1*D*). Thereby channel 2 triggers the laser (amplitude >3.3 V), as well as provides edges for timestamp detection.

### *In vivo* units recording

Animals were anesthetized with ketamine-xylazine (90/6 mg/kg, i.p.) and an additional injection of ketamine (45 mg/kg) if surgery required. The anesthetized mouse was placed on an electric thermal pad (37°C) and fixed into a stereotaxic frame with ear bars holding in front of and slightly above ears, on the temporal bone, to not block the ear canals. The skin over the vertex was removed and hydrogen peroxide (3%) was applied on the skull to visualize sutures. All coordinates were corrected to account for differences in the size of the head of each animal, similar to the virus injection procedure. Next, three small holes were drilled: at AP = −6.1 mm, ML = −2.3 mm (left DCN, for probe placement); at AP = −6.1 mm, ML = 2 mm (for optic fiber placement); and at AP = –2 mm, ML = 1 mm (for reference). Next, a micro screw was fixed in the reference coordinate using polymethyl methacrylate. The optic fiber was inserted into the brain using a micromanipulator positioned in a 45° angle to a depth of 5.58 mm, ending 0.5 mm away from the DCN. This angle avoids perturbing auditory pathways and gives appropriate space for the insertion of the recording electrode (NeuroNexus; single shank, 16 channels, 50 μm channel spacing, 177 μm^2^ recording site, 5 mm length). The recording electrode was dipped in fluorescent dye (1,1-dioctadecyl-3,3,3’,3’-tetra-methylindocarbocyanine perchlorate; DiI; Invitrogen) for 10 min before the procedure to visualize electrode placement *post hoc*. Three different recording depths were used (electrode tip at DV = −4.0 mm, −4.3 mm, or −4.5 mm). Unit responses were recorded under sound and/or light stimulation, with modalities presented at randomized order. Data acquisition was done using a headstage (Intan RHA2116 or Intan RHD2132; 16 unipolar channels) connected to a data acquisition board (Intan RHA2000 or Open-Ephys), at a sampling rate of 25 kHz (for experiments with Intan RHA) or 30 kHz (for experiments with Open-Ephys). The headstage reference and ground were separated in the headstage, then the ground was connected to the system ground and the reference was connected to the reference screw. Responses were visualized using Open-Ephys graphical user interface (Siegle et al., 2017). At the end of recordings animals were killed for histology.

### Unit analysis

Spikes were detected and clustered with a fourth order Butterworth digital bandpass filter (300–7500 Hz), negative spikes detected using a threshold from 2 to 4.5 SD, waveforms of 2 ms around the detected negative peak, and three features per channel. Peristimulus-time histograms (PSTHs) were calculated by counting occurrence of spikes in a time window of 100 ms around each transistor-transistor logic (TTL) (50 ms before and 50 ms after the TTL) and presented as mean number of spikes per time, where each bin corresponds to 2 ms. Units were classified as responding units as described by Parras et al. (2017). In brief, 1000 or 2000 PSTHs (depending on the number of trials played) were generated with random values in a Poisson distribution, with λ equal to the mean of values from the negative portion of the unit PSTH (baseline). Then, for real and simulated PSTHs, the mean of the negative PSTH values (baseline) was subtracted from the mean of the positive PSTH values (response), resulting in a baseline-corrected spike count. Finally, the *p* value was calculated as

(3) p=(g + 1)/(N + 1),

where *g* is the number of simulated histograms with corrected spike count bigger than the real unit spike count, and *N* is the total number of simulated histograms, which here is the number of trials presented at that unit recording. A cell was classified as responding to a stimulation if the resulting *p* value was <0.05. Cells were classified as responsive to sound only, light only, sound+light only, or sound and light. Spike rate was calculated as spike events per second along all the recording (including the stimulation period). The threshold of 9 Hz was considered to separate between slow-spiking and fast-spiking neurons, since ∼88% of neurons had firing rate <6.42 Hz and the remaining ∼12% had firing rate >9.24 Hz. Student’s *t* test, two-tailed, unequal variance was applied to compare firing rate between neurons and *p* values were Bonferroni-corrected when the same dataset was used for multiple comparisons. All firing rate values are represented as frequency ± SEM.

### Auditory brainstem responses (ABRs)

**Figure 2.**
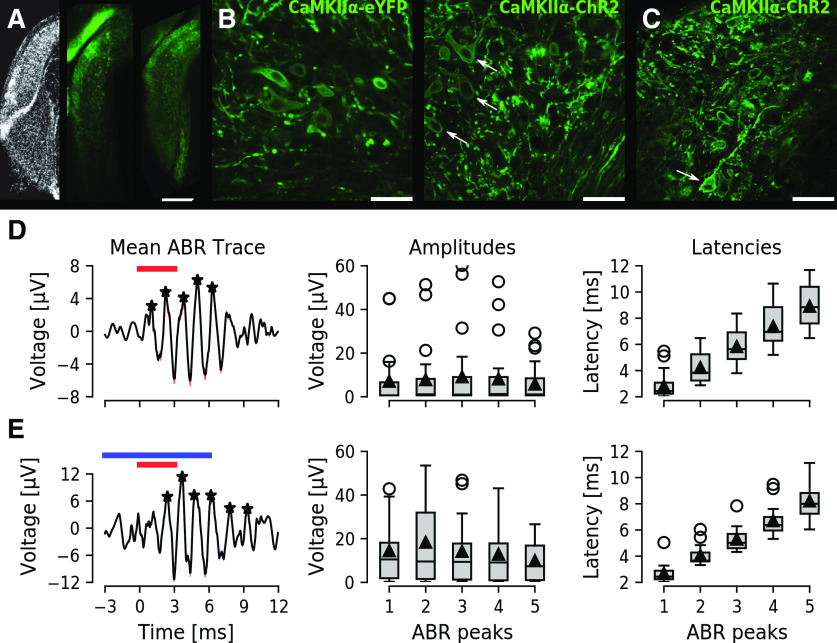
CaMKIIα-ChR2-eYFP-positive neurons in the DCN and ABR in injected animals. ***A***, Image of coronal brainstem sections with the DCN and VCN highlighted after DAPI nuclear staining (left), control CaMKIIα-eYFP (center), and CaMKIIα-ChR2-eYFP expression (right). Scale bar: 250 μm. ***B***, High-magnification confocal images showing several elongated horizontal somas (white arrows, possibly fusiform cells) labeled with membrane expression of eYFP. Scale bar: 40 μm. ***C***, Another high-magnification example of CaMKIIα-ChR2-eYFP labeling of the DCN. Two possible giant cells are in the deep layer (white arrow). Lateral is left and ventral is down for all images. Scale bar: 40* *μm. ***D***, ***E***, ABR waveforms recorded using electrodes lowered into the DCN in response to sound (***D***) and sound+light (***E***) stimulation protocols. Left, Mean (black line) and SEM (red shadow) ABR traces (*n* = 13), with detected peaks marked with black asterisks. Center, Box plots show group amplitude of the first five ABR peaks, horizontal lines show the median, triangles show mean, circles show outliers, whiskers bounding 99% of the data points. Right, Group latency of the first five ABR peaks.

ABRs are high-frequency event-related potentials that are commonly recorded from surface electrodes placed over the skin of the head or subdermally over the skull, but can also be recorded from auditory central areas (Lev and Sohmer, 1972; Achor and Starr, 1980), or even from brain areas unrelated to auditory processing (Jewett, 1970). Here, ABRs were extracted from the intracerebral electrode for recording extracellular spikes in the DCN. The high-frequency event-related local field potential was filtered (fourth order Butterworth digital bandpass filter from 500 to 1500 Hz), sliced (3 ms before and 12 ms after each sound pulse) and averaged, and ABR peaks were detected as a positive value 1 SD above the mean, larger than the previous value, and larger or equal to the next value. ABR peak values and latencies were then grouped for sound or light stimulation (see Fig. 2).

### Histology

Intracardial perfusion was conducted by deeply anesthetizing mice with ketamine/xylazine (180/12 mg/kg). Animals were fixed in a polystyrene plate, and a horizontal incision was made in the skin at the level of the diaphragm. Thoracic cavity was open by cutting the ribs laterally and the sternal medially. A 30G needle was inserted into the left ventricle for perfusion with cold PBS and an incision was made in the right atrium to allow for out-flow. In total, 20–30 ml of cold PBS followed by 20–30 ml of fixative (4% paraformaldehyde in 0.1 m phosphate buffer; pH 7.4) was used. Next, the brain was dissected and stored in 4% paraformaldehyde overnight. For free-floating vibratome (OTS-4000, EMS, Hatfield) sections the brain was stored in PBS before slicing; and for cryostat sections, the brain was kept in PBS with 30% sucrose until dehydrated (visualized by the brain sinking to the bottom of the solution), and frozen using isopentane at −60°C. Coronal sections (120 μm thick for free-floating, 30 μm for cryostat sections) of the brainstem, containing the DCN, were collected on glass slides and kept dark until examination of fluorescent expression by neurons. Cell nuclei were stained with 4’,6-diamidino-2-phenylindole (DAPI; Sigma) to visualize cell layers and borders of the DCN and VCN. Expression of optogenetic proteins was visualized by detection of genetically expressed enhanced yellow fluorescent protein (eYFP). Images were collected using Zeiss Observer Z1 fluorescence microscope or a Zeiss Examiner Z1 confocal microscope. The objectives N-Achroplan 5×/0.15; N-Achroplan 10×/0.25; Plan-Apochromat 20×/0.8; and Plan-Neochromat 40×/0.75 were used.

### CLARITY

The CLARITY procedure followed standard protocol and was previously described for another brain region (Hilscher et al., 2017). Data from the auditory brainstem was collected during the same experiment as previously published (Hilscher et al., 2017).

### Software availability

Our optogenetic and sound stimulation uses open systems and free open-source software. Recordings were done using Open-Ephys graphical user interface (Siegle et al., 2017). Calculations were done using Scipy (Jones et al., 2001) and Numpy (Van Der Walt et al., 2011), and all plots were produced using Matplotlib v2.2.4 (Hunter, 2007; Caswell et al., 2019). Spikes were detected and clustered using Klusta, and visual inspection was performed using Phy (Rossant et al., 2016). All scripts used for stimulation control and data analysis are available at https://gitlab.com/malfatti/SciScripts. Histology images were collected using AxioVision and Zen software, and edited for brightness and contrast in ImageJ (Schneider et al., 2012).

### Data availability

The datasets generated and/or analyzed in the current study are available on request.

## Results

### ABRs can be extracted during optogenetic excitation of CaMKIIα-ChR2-positive DCN neurons

We first tested whether the CaMKIIα promoter can be used to control subpopulations of DCN neurons. We injected viral vectors (rAAV5/CamK2-hChR(H134R)-eYFP or control rAAV5/CaMKIIa-eYFP) into the DCN for expression of ChR2 and/or eYFP under the control of the CaMKIIα promoter of one- to two-month-old C57Bl/6J mice. Four weeks later local protein expression in the DCN was examined, and showed strong eYFP signal in the vicinity of the injection site with a spread to both superficial and deeper layers of the DCN for both control and ChR2 vectors (Fig. 2*A*, center and right, respectively). eYFP showed strong membrane expression of soma and neurites of DCN neurons for both ChR2 and control constructs, especially in cells with elongated somas perpendicular to the DCN edge with thick basal dendrites spreading toward the molecular layer (possible fusiform cells; Fig. 2*B*, arrows). Smaller neuronal somas were also labeled with eYFP in the fusiform and deeper layers, as well as several large neuronal somas of the deep layer of the DCN with dendrites stretching along the internal edge of the DCN (possible giant cells; Fig. 2*C*, arrow). This shows that the CaMKIIα promoter is not specific for the DCN excitatory fusiform cell layer, although it seems to indeed label excitatory fusiform-like cells and giant cells with strong membrane expression.

To confirm that animals can hear the provided sound stimulation after injection of viral constructs we routinely extracted ABR waveforms from extracellular recordings. A high-impedance electrode (16 channel single shank silicon probe, Neuronexus), placed in the DCN for recording unit responses in anesthetized mice, was used to isolate ABRs in response to sound or concomitant sound and optogenetic stimulation. The anatomic correlation between auditory brainstem structures and ABR peaks, where I corresponds to the auditory nerve, II to cochlear nuclei, III to superior olivary complex, and IV and V to inferior colliculus (Henry, 1979) is useful for verifying an intact auditory brainstem system. We found all animals to display at least five peaks in the ABR waveforms (*n* = 13) in response to 80 dBSPL stimulation (Fig. 2*D*). Also, ABR mean amplitude and latency was not affected by concomitant sound and light stimulation (Fig. 2*E*). Together, this shows that the animals can hear the provided sound stimulus after viral vector injection procedure and during concomitant blue light stimulation.

### Optogenetic excitation of CaMKIIα-ChR2-positive DCN neurons is decreased by concomitant sound stimulation

**Figure 3.**
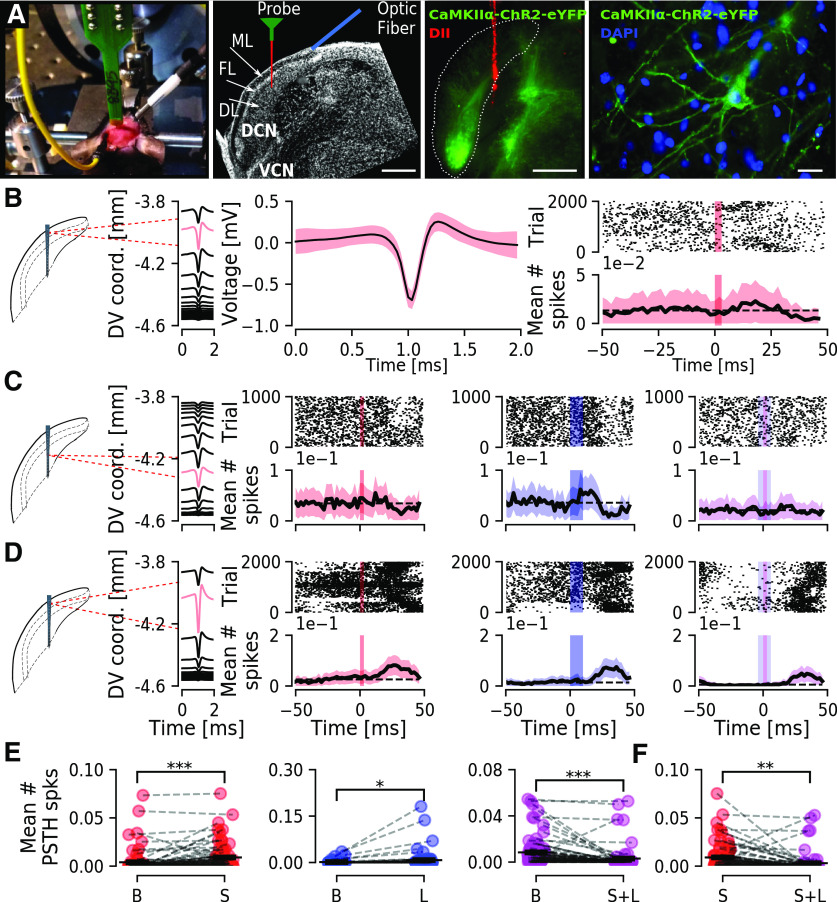
Activation of CaMKIIα-ChR2-eYFP expressing neurons in the DCN during sound stimulation can decrease unit firing. ***A***, left, Photography of a mouse in the recording setup and schematic representation of recording electrode and optic fiber for blue light stimulation in a DAPI labeled section showing the DCN sub-regions (ML-molecular layer, FL-fusiform cell layer, DL-deep layer). Center, Confocal image showing an example of eYFP expression in the dorsal region of the DCN with the probe tract colored by DiI. Scale bar: 100 μm. Right, Example of a DCN neuron expressing eYFP along the somatic membrane and proximal dendrites. Scale bar: 20 μm. ***B***, Example of a superficial unit (DV depth in mm) with its waveform shown, mean (black line), SEM (red shadow) at higher magnification (center), that responded to sound stimulation (red vertical bar) by increasing firing rate as seen by a PSTH. ***C***, Example of a deeper unit that does not respond to sound stimulation (left), increases firing in response to blue light stimulation (blue vertical bar, center), but shows no increase on both light and sound stimulation (right). ***D***, Example of a unit responding to sound (left), blue light (center) but with a slight decrease to concomitant sound and light stimulation (right). ***E***, Group mean number of spikes for all units (*n* = 76) showing a significant increase after sound (S) comparing to baseline (B; left) or blue light (L; center) stimulation (*p* = 5.3e-5 and 0.016) and a significant decrease after concomitant sound and light stimulation (S+L; right; *p* = 8.8e-5). ***F***, Group mean number of spikes for all units (*n* = 76) after sound is higher than after sound+light stimulation (*p* = 5.3e-4). *p < 0.05; **p < 0.005; ***p < 0.0005.

Next, we wanted to examine how DCN units respond to optogenetic modulations using the CaMKIIα promoter for expression of ChR2 within the DCN. Mice previously injected with CaMKIIα-ChR2 were anesthetized and fitted to a stereotaxic frame and a silicone 16-channel electrode was lowered vertically into the DCN (Fig. 3*A*). A total of 224 isolated units were identified, of which 148 were excluded for not responding to neither sound nor light stimulus (Table 1). From the remaining 76 units (*n* = 8 mice) 71% (54/76) responded to sound stimulation (3 ms, 80 dB, 5–15 kHz noise pulses presented at 10 Hz) and response to sound was quantified and visualized by PSTHs (Fig. 3*B*,*E*). Blue light stimulation (473 nm, 10 ms in duration, at 10 Hz with intensity of 5–7 mW/mm^2^ at fiber tip) delivered by a glass optic fiber to the DCN (Ø200 μm, inserted in a 45° angle from the contralateral side; Fig. 3*A*), elicited increased firing of units immediately following blue light stimulation (Fig. 3*C*,*E*). We found 25% (19/76) units responding to light stimulation. Out of these, 58% (11/19) responded exclusively to light stimulation, 26% (5/19) responded to either sound or light stimulation (Fig. 3*D*), and 16% (3/19) responded only to a combination of sound and light stimuli.

**Table 1:** Number of recorded units, separated by group, responding (sound and/or light stimuli), firing rate (low <9Hz, high >9Hz), and direction of modulation (down arrow decrease and up arrow increase firing rate on concomitant sound and light stimulation comparing to sound alone)

			Responding	Responding	Non-responding	Non-responding	Non-responding
	*n*	Responding	(low FR)	(high FR)		(low FR)	(high FR)
CaMKIIa-ChR2	224	76	65	11	148	78	70
			(↓ 34/↑ 31)	(↓ 7/↑ 4)		(↓ 26/↑ 52)	(↓ 37/↑ 33)
CaMKIIa-eArch3.0	86	17	16	1	69	46	23
			(↓ 4/↑ 12)	(↓ 1/↑ 0)		(↓ 31/↑ 15)	(↓ 12/↑ 11)
Chrna2-cre/	76	15	14	1	61	30	31
DIO-ChR2			(↓ 9/↑ 5)	(↓ 0/↑ 1)		(↓ 14/↑ 16)	(↓ 13/↑ 18)

The majority of units, 86% (65/76), had firing rates from 0.05∼6.46 Hz (average 1.5 ± 0.19) while a smaller proportion of units recorded had higher firing rates (14%; 11/76), from 8.83 ∼72.43 Hz (average 26.76 ± 5.81 Hz; Table 1). Including all 76 responding units, averaged over 1.5 min including stimulus epochs, and comparing to specific stimuli showed a significant increase in response to sound (*p* = 5.3e-5; Fig. 3*E*, left) or light (*p* = 0.016; Fig. 3*E*, center) but, interestingly, a decrease in response to concomitant sound and light stimulation (*p* = 8.8e-5; Fig. 3*E*, right). Also, the mean number of spikes in response to sound were significantly higher than to concomitant sound and light (*p* = 5.3e-4; Fig. 3*F*). This shows that optogenetic excitation using ChR2 can increase firing of DCN units, even in units not directly responding to sound or light, but controversially, presence of simple sounds during optogenetic stimulation can decrease the overall unit firing.

### Inhibiting CaMKIIα-eArch3.0-positive neurons in the DCN delay response to sound and bidirectionally affect units not responding to sound

**Figure 4.**
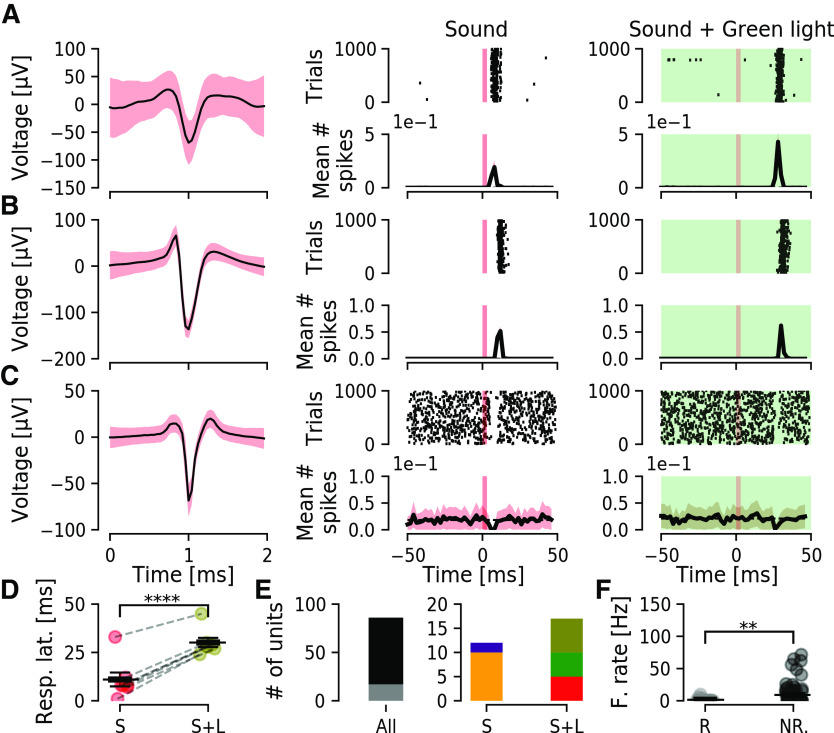
Inhibition of CaMKIIα-eArch3.0 expressing neurons can delay unit sound responses. ***A***, Example of a unit (mean, black line; SEM, red shadow) that responds to sound, while during inhibition of CaMKIIα-eArch3.0-positive DCN cells using green light (5- to 7-mW/mm^2^ laser intensity, green background) this unit shows delayed excitation in response to sound. ***B***, Another unit showing time-locked excitation in response to sound stimulation. This excitation is delayed by ∼20 ms when CaMKIIα-eArch3.0-positive DCN cells are inhibited by green light. ***C***, Example of a DCN unit with a negative response following sound stimulation. This pause in firing is delayed by CaMKIIα-eArch3.0-positive DCN cells inhibition using green light. ***D***, Group latency in response to sound is significantly increased for all units responding to both stimulation (*n* = 7, *p* = 6.7e-6). ***E***, Quantification of numbers of units. Left, Out of 86 units (black), 17 (gray; 20%) responded to the provided stimulation. Right, Out of 12 units responding to sound, 10 (orange; 83%) increased and two (purple; 17%) decreased firing in response to sound. Out of 17 responding units, five (red, 29%) responded only to sound (S), five (green, 29%) responded only to sound and light combined (S+L), and seven (olive, 41%) responded to both stimulations. ***F***, Responding (R) units showed lower firing rate than non-responding (NR) units (*p* = 5.8e-4). **p < 0.005; ****p < 0.00005.

We then tested whether we could inhibit CaMKIIα-eArch3.0-positive DCN neuron response to sound using the outward proton pump eArch3.0. We injected adult (one month) wild-type C57Bl/6J mice unilaterally with Archaerhodopsin-containing viral vectors (CaMKIIα-eArch3.0-eYFP) and four weeks later extracellular activity in the DCN was recorded. Units were recorded in response to short sound pulses (3 ms, 80 dB, 5∼15-kHz noise pulses presented at 10 Hz) and next using an optic fiber coupled to a green laser source (543 nm excitation) we examined whether responses to sound could be abolished by concurrent green light stimulation (543 nm, 20 s, repeated 5× with 10 s interval, so it is concomitant to sound pulses). Out of 86 units isolated (*n* = 4 mice), we found that 17/86 (20%) units responded to stimulation (Fig. 4*D*, left), from which 12/17 (71%) responded to sound stimulation (Fig. 4*E*, left). The most striking finding was that instead of inhibition of responses to sound we found several types of unit responses being delayed during green light stimulation (Fig. 4*A*,*B*); 7/17 (41%) units that sharply responded to sound showed a delayed response to sound with mean latency of 19.1 ± 1.22 ms when the DCN CaMKIIα-eArch3.0 cells were inhibited during sound stimulation (Fig. 4*A–C*,*E*, right). The delayed response to sound continued strongly time-locked. This could suggest that some DCN CaMKIIα-eArch3.0-positive neurons may be inhibitory, as green light stimulation could involve disinhibition of cartwheel cells, and complex spiking units are known to generate delayed responses in PSTH in the range of 10 to 40 ms delay (Parham et al., 2000). Examining responses from the units isolated, the majority (10/12) of units responded to sound stimulation by increasing firing rate (Fig. 4*A*,*B*), while 2/12 units decreased the firing rate in response to sound stimulation (Fig. 4*C*) and both such responses were delayed in the presence of green light stimulation. Furthermore, 5/17 units responded exclusively to sound, while another 5/17 units responded exclusively to the combination of sound and green light (Fig. 4*D*, right).

**Figure 5.**
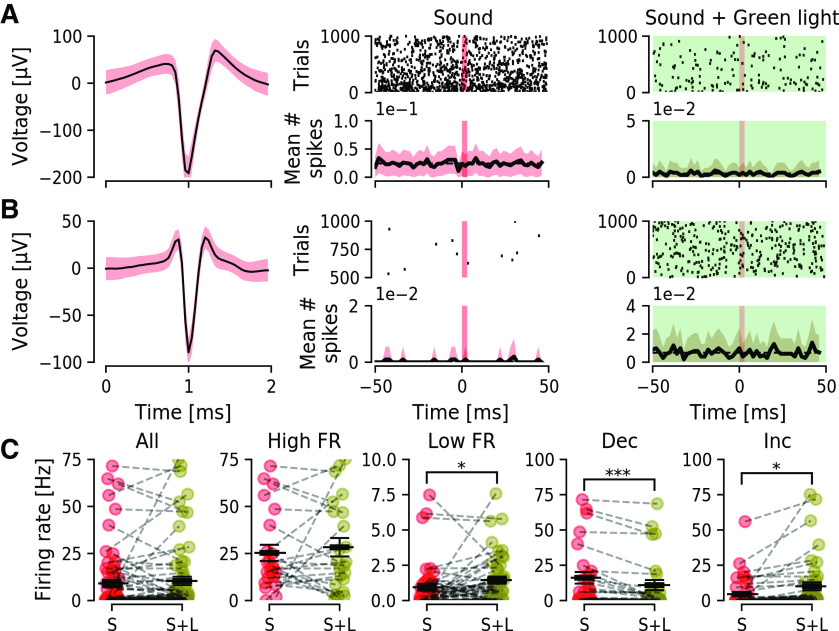
Inhibition of CaMKIIα-eArch3.0-positive cells in the DCN can both increase and decrease excitation of DCN units not responding directly to sound. ***A***, DCN unit (−3.8 mm DV) showing high spontaneous firing but not responding directly to sound (center) showing decreased firing rate on green light stimulation (inhibiting CaMKIIα-eArch3.0-positive DCN cells, right). ***B***, Unit at similar depth as ***A*** showing increased firing rate on inhibition of CaMKIIα-eArch3.0-positive DCN cells, but no response directly to sound. ***C***, Group firing rate of all units not responding directly to sound (*n* = 69) in the presence of sound (red) or concomitant sound and green light (olive). Units were divided into high and low firing rate, and a significant increase in firing was found for low firing units (*p* = 0.03). Units were also divided into units that decrease or increase firing rate comparing both stimulations, and a significant decrease and increase was found (*p* = 4.7e-4 and 0.01, respectively). *p < 0.05; ***p < 0.0005.

Examining firing rates of sound responding units and comparing to non-responding units showed that we targeted slow firing units responding to sound (1.5 ± 0.6 and 9.1 ± 2 Hz for responding and non-responding units, respectively). Still, it is also important to examine the effect of DCN inhibition on units not responding directly to sound. We found, for the remaining 69/86 units that did not respond to sound stimulation, 39% (27/69) of units to decrease spontaneous firing rate under green light stimulation (Fig. 5*A*,*C*). Out of the 27 units, 12 were high-frequency firing (33.51 ± 6.3 Hz) that decreased firing to 70% of the initial frequency (23 ± 6.68 Hz) under green light stimulation, while 15 units were low-frequency firing (2.19 ± 0.57 Hz), decreased firing frequency by 58% (1.26 ± 0.48 Hz) on green light stimulation. On the contrary, 42 units increased firing frequency on green light stimulation (Fig. 5*B*,*C*), where 11 high-frequency firing units increase in firing frequency to ∼double the initial frequency (16.4 ± 4.38 to 34.11 ± 6.76 Hz) while 31 low firing units on average increased firing from 0.33 ± 0.11 to 1.54 ± 0.29 Hz (Fig. 5*B*,*C*). Overall, non-responding units that had low firing rates in response to sound showed a significant increase in firing rate (*n* = 46/69 units, *p* = 0.03). Also, CaMKIIα-eArch3.0 inhibition caused a bidirectional effect, with 27/69 decreasing (*p* = 4e-4) and 42/69 increasing (*p* = 0.01) firing rate (Fig. 5*C*). This highlights the complexity of the DCN and how precaution must be taken when attempting to decrease neuronal activity *in vivo* of the auditory brainstem using tools such as CaMKIIα-eArch3.0.

### The Chrna2-cre transgenic line targets putative T-stellate cells and bushy cells of the VCN

**Figure 6.**
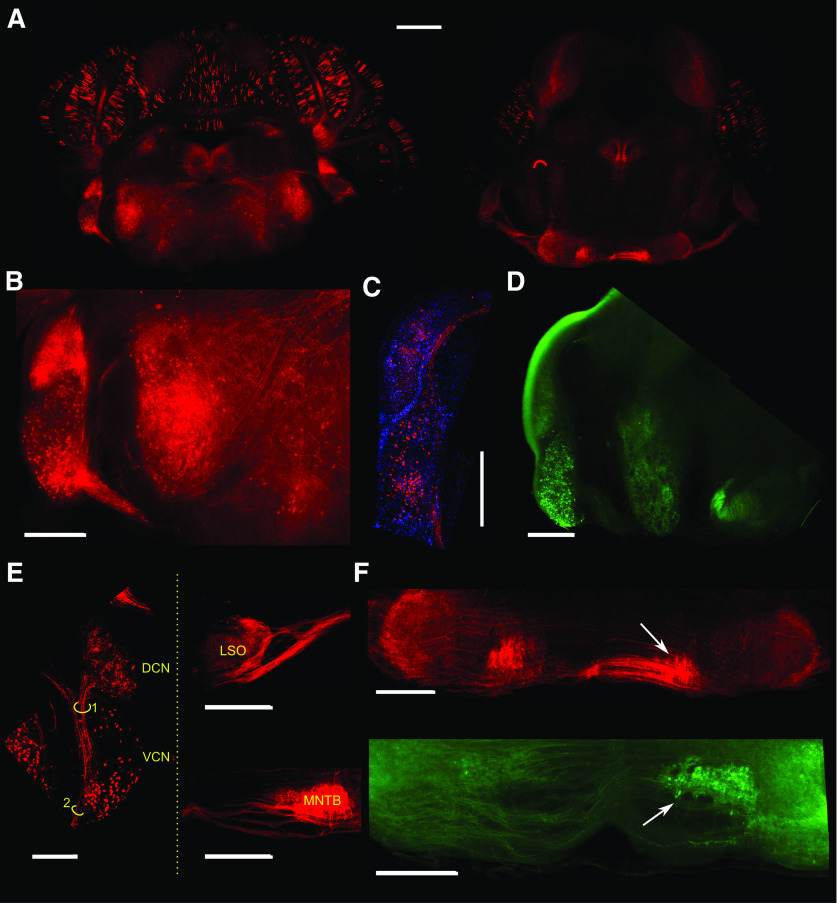
Confocal images showing tdTomato expression and Chrna2-cre/DIO-ChR2-eYFP expression in transversal brainstem sections from Chrna2^cre^/tdTomato^lox^ mice. ***A***, Mosaic of images showing a coronal overview of Chrna2-tdTomato expression in the cochlear nucleus (left) and MNTB (right) AP coordinate. ***B***, Zoom-in image showing tdTomato expression in the VCN and DCN. Red cell bodies can be clearly seen in the VCN area while red expression is more diffuse in the DCN, suggesting this area is showing dense axonal terminations from VCN T-stellate cells. ***C***, Another example showing Chrna2-tdTomato expression overlayed with DAPI staining. ***D***, Image showing unilateral expression of eYFP following local injections with cre-dependent ChR2 (Chrna2-cre/DIO-ChR2-eYFP) constructs in the VCN. The VCN contains strongly labeled cell bodies and the DCN shows diffuse green labeling. The strong green edge of the DCN is an artifact of the mounting medium. The ipsilateral S-shaped LSO is also strongly labeled by eYFP. ***E***, Image of a coronal slice from the CLARITY dataset of a Chrna2cre^lox^ mice showing the cell bodies in the VCN and projections going up to the DCN. Highlighted bundles project to LSO (1) and MNTB (2). ***F***, top, Zoom-in showing strong labeling of the MNTB and the lateral superior olive (LSO; arrow) suggests that also anteroventral VCN bushy cells are expressing the Chrna2 promoter. Bottom, Subsequent brainstem section (from the same injected animal from ***D*** showing strong labeling of the contralateral MNTB (arrow). Scale bars: 1 mm (***A***) and 500 μm (***B–F***).

**Figure 7.**
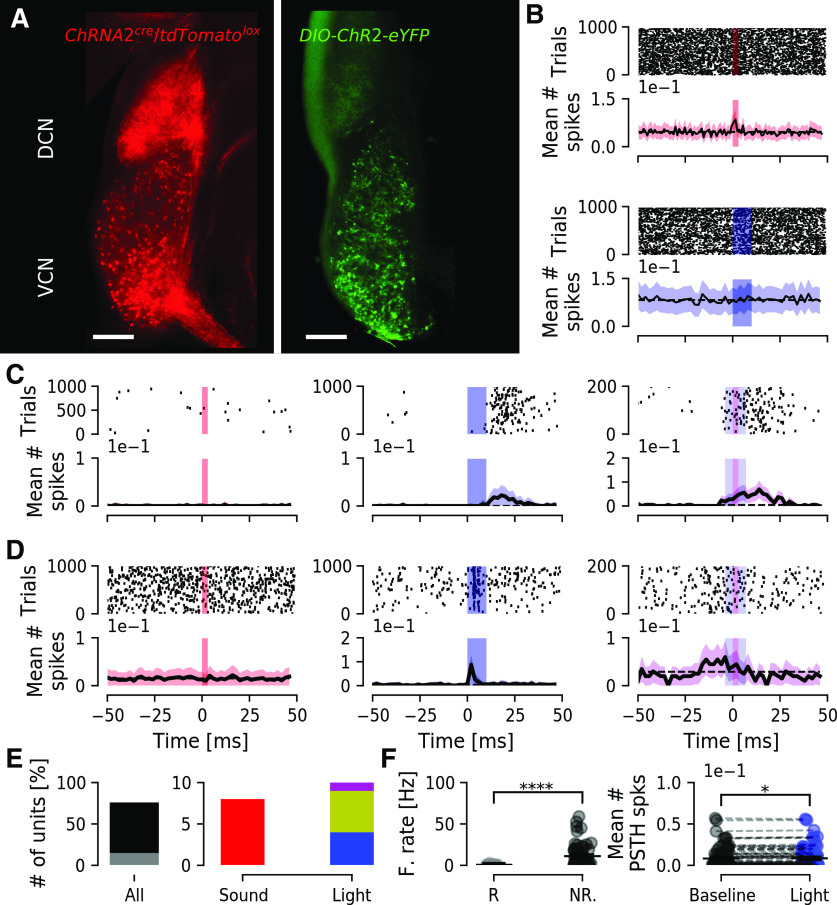
Chrna2-cre-positive neurons of the VCN can be targeted to drive activity of DCN neurons. ***A***, left, Confocal image showing tdTomato (red) expression in VCN cell bodies and strong axonal arborizations in the DCN fusiform and deep layers. Right, Expression of ChR2-eYFP (green) in Chrna2-cre-positive neurons of the VCN, with diffuse green innervation of the DCN. ***B***, Example of a unit from the DCN that responds to sound but not light stimulation. Line PSTH bin size is 1 ms for sound and 2 ms for light responses. ***C***, Example of a unit that does not respond to sound stimulation, but responds to light stimulation (center). In 200 trials of combined light and sound stimulation the unit responds to both stimuli, with what appears as an anticipation of sound. ***D***, Example of a unit not responding to sound pulses but responds with high fidelity to light stimulation of the VCN. When sound and light stimuli were combined, the unit failed to respond. ***E***, Quantification of number of units according to response. Left, Out of 76 units, 15 (gray) responded to stimulation. Right, Out of 15 responding units, eight (red; 53%) responded to sound, four (blue; 27%) responded only to light, five (yellow, 33%) responded to both sound and light, and only one (magenta, 7%) responded to light or concomitant sound and light stimulation. ***F***, left, Group firing rate of responding (R) units show lower firing than non-responding (NR) units (*p* = 2.6e-7). Right, Group mean number of spikes for all DCN units, showing a significant increase after light stimulation of the VCN circuit (black, baseline; blue, light stimulation; *n* = 76 units, *p* = 0.026). *p < 0.05; ****p < 0.00005.

A recent and interesting transgenic cre-line is the chrna2-cre mouse that targets specific interneuron populations in different brain and spinal cord regions (Leão et al., 2012; Mikulovic et al., 2015; Perry et al., 2015; Hilscher et al., 2017; Siwani et al., 2018). So far there are no reports of Chrna2-cre expression in auditory areas, so here we start by exploring Chrna2-cre expression in the cochlear nucleus and superior olivary complex. Heterozygous Chrna2-cre transgenic mice were crossed with homozygous tdTomato-lox reporter mice to visualize Chrna2+ cells in the auditory brainstem (Figs. 6, 7*A*). We found Chrna2+ cells in the VCN, with dense projections of axons branching into the DCN as well as to the superior olivary complex (Figs. 6, 7*A*). Brains processed for CLARITY examination (Hilscher et al., 2017) also show Chrna2+ VCN cells with projections to the DCN (Fig. 6*E*). In order to identify the boundaries of DCN and VCN and examine any soma labeling in the DCN, slices of Chrna2-tdTomato animals were co-stained with DAPI (Fig. 6*C*). Very few red labeled somas were identified in the DCN of Chrna2-tdTomato animals or in CLARITY images and did not relate to any particular region (Fig. 6*C*,*E*). Next, Chrna2-cre mice were injected with cre-dependent ChR2 (Chrna2-cre/DIO-ChR2-eYFP) viral vector into the VCN, and the expression pattern was similar to the Chrna2-tdTomato (Fig. 6*D*). Based on projection patterns we assume Chrna2+ cells of the VCN to comprise of both stellate and bushy cell subtypes (Fig. 6, 7*A*). Previously, T-stellate cells have been shown to respond to cholinergic agonists (have acetylcholine receptors), while D-stellate cells are insensitive to carbachol (Fujino and Oertel, 2001); therefore, Chrna2-cre-positive neurons projecting to the DCN (Fig. 6*E*) are most likely T-stellate cells (Oertel et al., 2011). T-stellate cells also project to the ipsilateral LSO (Oertel et al., 2011), which supports the strong labeling of ChR2 in the ipsilateral LSO (Fig. 6*D*). The ipsilateral LSO is also labeled by VCN bushy cells as projections to the contralateral medial nucleus of the trapezoid body (MNTB; Fig. 6*F*) as well as the ipsilateral LSO were apparent, thereby indicating that Chrna2-cre labels both globular and spherical bushy cells.

### VCN Chrna2+ neurons can generate indirect optogenetic activation of DCN neurons

To investigate whether optogenetic control of putative T-stellate cells can excite DCN neurons, Chrna2-cre mice were unilaterally injected with cre-dependent ChR2 (Chrna2-cre/DIO-ChR2-eYFP) into the VCN generating labeling of diffuse fibers spreading in the deep layers of the DCN (Fig. 7*A*). To investigate whether DCN cells can be indirectly excited by light stimulation of ChR2-eYFP expressing Chrna2+ cells of the VCN, we recorded unit responses in the DCN from anesthetized mice while stimulating with blue light (10 ms, 473 nm light pulses at 10 Hz) with an optic fiber placed in a 45° angle into the VCN. We first analyzed whether units responded to sound stimulation. Out of 76 units extracted, only 15 units (*n* = 8, 6, and 1, from three mice, respectively) responded to sound and/or light stimulation (Fig. 7*B*,*E*). For units not responding to sound, we found examples of a DCN unit with response to VCN light stimulation that was prolonged and appeared increased in the presence of sound (Fig. 7*C*). Also, one DCN unit with highly temporally precise responses to VCN light showed a loss of response in the presence of sound (Fig. 7*D*). In summary, 4/15 units responded only to light stimulation, 2/15 units responded only to sound stimulation (Fig. 7*B*,*E*), and 13/15 units responded to light or combined light and sound stimulation (Fig. 7*C*,*E*). Excitation of VCN Chrna2+ cells significantly increased firing rate in DCN units, both responding and not responding to sound (Fig. 7*F*). Still, the identity of these different units has to be further investigated but highlights that the Chrna2-cre line has potentials for auditory research.

### Light stimulation alone does not alter the DCN circuit response to sound

**Figure 8.**
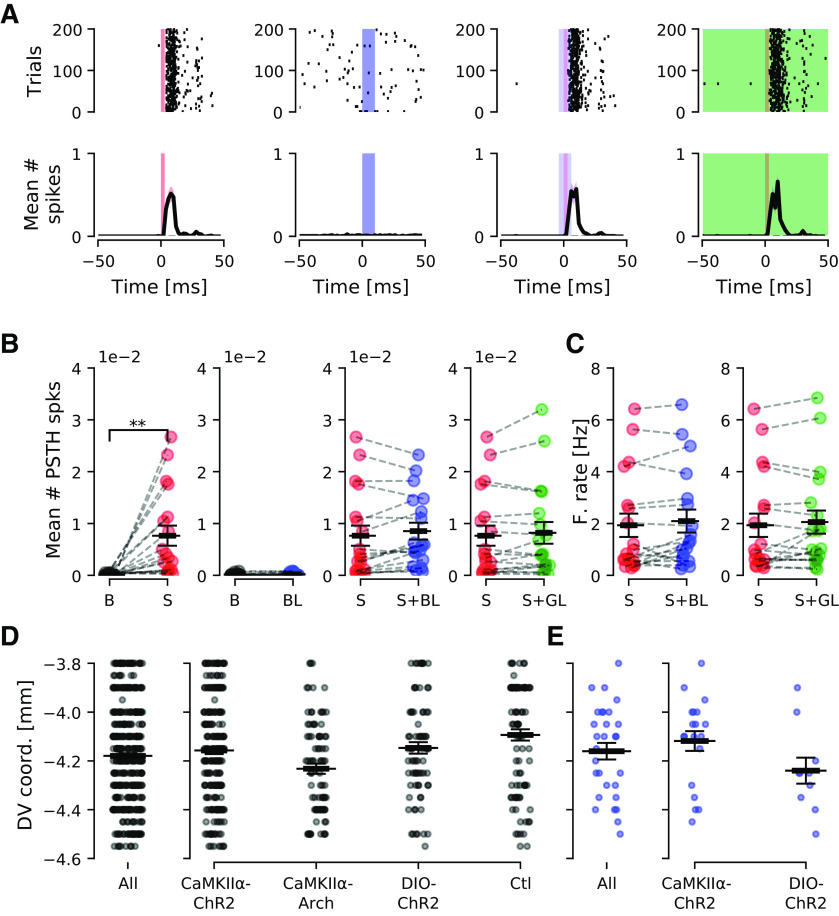
Blue and green light (5–7 mW/mm^2^) do not alter DCN unit firing in response to sound in control animals. ***A***, Example of a DCN unit showing distinct onset response to sound (left, 200 repetitions shown), no response to blue light pulses (200 pulses, 10 ms each, presented at 10 Hz), and no change in the sound response with concomitant, continuous blue (10 ms) or green light stimulation (20 s). ***B***, Group data (*n* = 18 units, 5 animals) showing mean number of PSTH spikes increasing from baseline (***B***) firing to sound responses (S; *p* = 4.9e-3), and no response to blue light simulation (BL, center). Sound responses were not altered by concomitant continuous blue or green light (GL) stimulation. ***C***, Firing rate was not significantly altered by blue or green light stimulation. ***D***, DV coordinates for all recorded units from all groups (left) and separated for each group (right). ***E***, DV coordinates for all units responding to light stimulation (left), separated for each group (right). The CaMKIIα-eArch3.0 group was not included, since there was no light-only stimulation. **p < 0.005.

As recent work has shown that optogenetic light stimulation heats up tissue and many physiological responses are heat sensitive (Owen et al., 2019), it is important to confirm that our findings are not because of local heating of the DCN or VCN. Therefore, we tested both the blue and green light stimulation in control, uninjected mice (*n* = 5). The control experiments (*n* = 94 units of which 18 units responded to sound stimulation) showed no significant change in overall number of spikes in response to blue light pulses (Fig. 8*A*,*B*), thereby not affecting the sound responses. Sound pulses during green light stimulation protocols showed no change in mean number of spikes (Fig. 8*A*,*B*) nor changes in firing frequency in response to concomitant 20 s green light stimulation (Fig. 8*C*). Also the depth of light penetration is important to take into consideration (Ash et al., 2017) for studying circuit output. Depth profile plots of all recorded units (Fig. 8*D*) and of units responding to light (Fig. 8*E*) show that units recorded responding to light pulses are spread across the DCN DV axis. This suggests that the light penetration was sufficient to reach a large portion of the DCN. Also, this supports the conclusion that different cell types are targeted by the CaMKIIα promoter as different cell types of the DCN are organized in different layers. Similarly, Chrna2+ VCN cell light activation, with the fiber targeting the VCN region, triggered activity at different depths of the DV axis of the DCN (Fig. 8*E*). Thereby the Chrna2+ (putative T-stellate cells) cell axons might target a large area of the DCN as also suggested by CLARITY images.

## Discussion

There is a large variety of genetic tools for dissecting the role of specific neuronal populations, such as transgenic mice expressing a reporter protein or a recombinase, or viral vectors with different promoters for certain cell specificity. Currently widely used viral vector/promoter-based gene expression in neuroscience are hSyn (synapsin) for all neurons, CaMKIIα for cortical pyramidal cells, mDLx for interneurons, GFAP for glia and a cohort of synthetic (any cell) promoters. In regard to transgenic mice (e.g., expressing cre recombinase under the control of specific gene expression), there is a large library of strains for targeting specific interneuron populations in the cortex or single neurotransmitter systems in subcortical nuclei like SERT-cre (serotonergic), DAT-cre (dopaminergic), Chat-cre (cholinergic) etc. However, there is, to our knowledge, no Cre line or promoter-based vector for specific tagging of auditory neurons, especially those residing in the brainstem.

In this work, we first show that the CaMKIIα promoter targets DCN neurons of different morphology, and at different depths of the DCN, and thereby not only putative fusiform cells/fusiform cell layer. Next, we show that excitation of CaMKIIα-ChR2-positive DCN cells during sound stimulation generates normal ABRs, and does not disrupt hearing pathways. Still, we show that optogenetic excitation of CaMKIIα-ChR2-positive DCN cells modulates DCN unit firing rate, and that such light stimulation is sensitive to concurrent sound stimulation. When aiming to inhibit DCN activity we found that inhibiting CaMKIIα-eArch3.0-positive DCN cells can delay response to sound instead of decreasing DCN units firing rate. More so, in units not responding directly to sound, CaMKIIα-eArch3.0 inhibition of the circuit could bidirectionally alter firing rate of units. Lastly, we show that the Chrna2-cre line strongly labels cell bodies in the VCN and that these cells are putative T-stellate cells, based on pharmacology and projection patterns (Oertel et al., 2011), and bushy cells, based on innervation to the MNTB and LSO. Furthermore, Chrna2-cre/DIO-ChR2 excitation in the VCN increased firing of a small number of DCN units and this excitation appeared temporally precise. Still, this connectivity needs to be further characterized both presynaptically and postsynaptically.

Our experiments were performed in C57BL/6J mice as these animals are commonly used for genetic manipulations with optogenetic tools. It is known that the C57 mouse strain has a point mutation in the *cdh23* gene (Noben-Trauth et al., 2003) and thereby suffers a progressive high-frequency hearing loss after approximately three months of age. Therefore, we performed all experiments in mice around two months of age, with viral constructs injected at one month of age and allowed three to four weeks for adequate protein expression before optogenetic experiments. Still, it is important to confirm that animals indeed can hear the tested frequencies used in experiments. Here, extracted ABRs showed typical ABR peaks indicating neuronal responses to sound at several levels of the auditory brainstem, and thereby intact hearing of tested stimuli. Since extracellular recordings can pick up ABR signals, ABR protocols can be useful to routinely add to single or multiunit recordings in the DCN especially when using older mice on a c57BL/6J background. Recently, new cre-lines expressing channelrhodopsin on a CBA background, with preserved hearing throughout adult life, have been developed (Lyngholm and Sakata, 2019). Lyngholm and Sakata (2019) show that CBA mice expressing ChR2 coupled to the parvalbumin promoter could excite cortical narrow spiking neurons (inhibitory interneurons) on light stimulation, and inhibit broad spiking units as effectively as in mice with a C57 background. On the other hand, a benefit of using the c57BL/6J mouse line is that it is more susceptible to acoustic overexposure than other strains (Willott and Erway, 1998; Davis et al., 2001) and thereby a suitable animal model for studying noise-induced tinnitus.

While several studies have applied general promoters to achieve optogenetic control of the DCN (Shimano et al., 2013; Darrow et al., 2015; Hight et al., 2015) there is still a lack of studies showing subpopulation control of the DCN *in vivo*. The CaMKIIα promoter is often used for targeting excitatory neurons in the neocortex and hippocampus (Wang et al., 2013), but here, we found that the CaMKIIα promoter, for the viral constructs tested, would target morphologically different cell types within the DCN. This is in agreement with studies of the olfactory bulb, where CaMKIIα-GFP-positive neurons co-localize with GABA immunoreactivity (Wang et al., 2013). Still, to decrease activity of DCN excitatory neurons would be highly interesting for alleviating tinnitus. In slice preparations, vesicular glutamate transporter 2 (VGluT2) transgenic mice have already been used for targeting DCN fusiform and stellate cells to investigate control of DCN fusiform cell activity (Apostolides and Trussell, 2013a). Inhibitory DCN activity has been investigated using glycine transporter 2 (GlyT2-cre mice) for controlling DCN cartwheel cell firing *in vitro* (Apostolides and Trussell, 2013b; Lu and Trussell, 2016). Also the GABA/glycine transporter (VGAT) promoter has been used to excite inhibitory interneurons to study inhibitory neurotransmission in slices of the VCN (Xie and Manis, 2014), for example.

Attempting to silence DCN units responding to sound, using light stimulation of CaMKIIα-eArch3.0 expressing neurons showed that units could be inhibited using eArch3.0, but also that green light exposure generated a distinct delay in response-onset to sound. The delay was consistently around 20 ms suggesting polysynaptic activity, possibly from the recruitment of complex spiking cartwheel cells that can respond with 20 ms delay to pure tone stimulation (Parham et al., 2000). Also PSTH of unidentified neurons with a 40 ms delay between initial and basal firing have been reported for guinea pigs (Robertson and Mulders, 2018). Our experiment could not identify the type of unit responsible for this delay, but it shows that silencing CaMKIIα-eArch3.0 expressing neurons is not enough to disrupt sound generating activity in the DCN circuit. Some studies have pointed to technical problems when using the proton pump eArch3.0, as it may affect intracellular pH of presynaptic membranes and promote neurotransmitter release if light is applied continuously for several minutes (Mahn et al., 2016). Mahn et al. (2016) showed that 5 min of continuous eArch3.0 activity significantly increased the EPSC rate (Mahn et al., 2016). Thereby, eArch3.0 may not be the most appropriate tool for inhibiting neurons of the DCN for longer time periods. Here, we found that applying green light stimulation in blocks of 20 s was sufficient to alter temporal coding of some units and silence others. An advantage of green light stimulation, compared with blue, is that green wavelength light can penetrate deeper into tissue without being scattered. For example, it has been shown in a modeling study that green light penetrates skin tissue twice as deep as blue light (Ash et al., 2017). Thereby, the green light applied here should be adequate for illuminating the DCN, and not be the reason for failed neuronal silencing in units showing delayed sound response. Still, the placement of the recording electrode could influence our findings as we are only sampling local neurons according to the probe location. As we describe in methods, we adjusted our coordinates to the animal skull size and recorded at three different depths to cover an as large as possible region of the DCN for each animal, without inserting the probe at multiple ML/AP locations or aspirating the cerebellum, as done in other rodent studies (Kaltenbach and Zhang, 2007; Shore et al., 2008; Finlayson and Kaltenbach, 2009; Koehler et al., 2011; Dehmel et al., 2012; Manzoor et al., 2012). Furthermore, spontaneously firing neurons that decreased or increased firing on green light stimulation were recorded all along the probe, showing that they were not from any specific DCN layer. An interesting finding from CaMKIIα-eArch3.0 experiments was that many neurons not responding directly to brief sound of 5–15 kHz were indirectly affected by silencing CaMKIIα-eArch3.0-positive neurons in the DCN. Also, that inhibiting neurons expressing CaMKIIα-eArch3.0 can be used to modulate both high-frequency and low-frequency firing neurons not responding directly to sound, and that this modulation was bidirectional. This suggests that CaMKIIα expressing neurons can modulate excitation/inhibition ratios to some extent (see Fig. 5*C*). Here, stimulating the DCN with longer sound at additional frequencies would clarify what type of units respond by decreasing or increasing spike activity when inhibiting CaMKIIα+ DCN neurons.

One of the disadvantages of optogenetic stimulation is that high intensity laser stimulation can increase the temperature of the surrounding tissue and that many neuronal responses are temperature-dependent (Owen et al., 2019). Especially 15 mW/mm^2^ green light for 1–5 s can alter firing rate and generate heat-sensitive ion currents (Owen et al., 2019). Here, at the light intensity of 5–7 mW/mm^2^, we did not observe altered firing rate to sound in the presence of blue light pulses or 20 s green light stimulation in control animals, indicating that the changes we observed in the injected animals were attributed to the expression and activation of opsins.

A limitation of our study is that we did not assess the best frequency of units. Thereby the sound stimulus will not display full firing potential nor specific firing patterns (such as pauser, onset, build-up units). However, as light stimulation also was brief, it allows for more direct comparison between modulation of unit activity in responses to sound or after exciting DCN units with light. By not assessing the best frequency and not presenting sound of different frequencies at different intensities also prevents us from building frequency response areas (Palmer et al., 2013) for each unit. However, the linear disposition of the channels in the probe allowed recording of units along all the DCN layers, which is known to aggregate different types of cells (Oertel and Young, 2004). One of the interesting findings of this study was that sound pulses alone elicited more spikes than sound+light stimuli. There are different possible explanations for this, such as inhibitory neurons express opsins and thus the observed decrease could be part of motifs of feed-forward inhibition (Roberts and Trussell, 2010). Or, the fact that the light stimulus is slightly longer and starts earlier than the sound pulse, thereby the light pulse could immediately activate inhibitory neurons just before the sound pulse, which would decrease the probability of the circuitry to be activated compared with the sound pulse alone. Also, for excitatory neurons expressing ChR2, the unit could be activated by the light pulse, and next be in a non-excitable state when the sound pulse is presented. This “light-on-sound” masking was shown before (Hernandez et al., 2014), where expressing ChR2 in cochlear spiral ganglion neurons and presenting the sound stimulus embedded in blue light stimulation masked the evoked ABR, although not completely abolished (Hernandez et al., 2014).

Our work also shows for the first time that the Chrna2-cre line targets two different cell populations of the VCN; both putative T-stellate cells and bushy cells of the VCN. T-stellate cells, also called Type I/multipolar/choppers, are excitatory neurons of the VCN with diverse connectivity but project mainly to the contralateral inferior colliculus (for review, see Oertel et al., 2011). T-stellate cells have been shown to have axon collaterals that go into the deep layers of DCN without reaching the fusiform cell layer (Oertel et al., 2011) which is similar to the diffuse axon terminals we observed (Fig. 7*A*; and CLARITY data). Our virus labeling also corroborates the indication that T-stellate cells provide input to the ipsilateral LSO, as we observed strong labeling of the ipsilateral LSO. However, bushy cells of the AVCN also project to the ipsilateral LSO, making additional experiments needed to confirm the degree of such connectivity. It is also known that T-stellate cells are modulated by nicotinergic agonists while D-stellate cells are not (Fujino and Oertel, 2001), in agreement with our cre-line that specifically targets neurons with nicotinic receptors containing the Chrna2. Recently, T-stellate cells were shown to innervate neighboring T-stellate cells responding to similar sound frequencies, regulating local excitation through nitric oxide-dependent plasticity mechanisms (Cao et al., 2019). The Chrna2-cre line could possibly aid in studies of hyperexcitability in T-stellate cells relating to hearing loss and/or tinnitus (Coomber et al., 2014; Cao et al., 2019). Here, using Chrna2-cre mice and cre-dependent viral constructs we could excite DCN units that did not respond directly to sound, but responded temporally precisely to light stimulation of the VCN, suggesting that specific circuits may be targeted using these animals. We found no disruption of hearing on optogenetic stimulation, but specifically altered network activity compared with brief sound stimulation, including delayed responses and disinhibition of activity. Interestingly, we also found altered spiking activity for units not responding directly to sound. Furthermore, stimulation of bushy cells may especially be useful for studies of the calyx of Held presynaptic release and/or sound localization studies using the Chrna2-cre line. Together, these results open up for more detailed control of cochlear nucleus circuit output *in vivo* and novel tools for studying tinnitus mechanisms.

In conclusion, optogenetic stimulation of CaMKIIα+ DCN neurons or Chrna2 expressing VCN neurons can be used to manipulate unit firing of the DCN and to modulate response to sound. We found that the CaMKIIα promoter can be used to optogenetically lower or scramble timing of neuronal activity of the DCN but not to specifically activate the DCN in a temporally precise manner or increase response to sound. As the DCN is implicated in tinnitus, the CaMKIIα promoter could provide a useful, low-cost (compared with transgenic animals) option for lowering overall DCN activity. Furthermore, the Chrna2 promoter provides an interesting genetic tool as smaller and more specific network activity modulation may be achieved compared with sound stimulation.

## Synthesis

Reviewing Editor: Leonard Maler, University of Ottawa Decisions are customarily a result of the Reviewing Editor and the peer reviewers coming together and discussing their recommendations until a consensus is reached. When revisions are invited, a fact-based synthesis statement explaining their decision and outlining what is needed to prepare a revision will be listed below. The following reviewer(s) agreed to reveal their identity: NONE.

Editorial comments

The manuscript provides a useful technical advance by testing the effects of expression of CaMKIIa and channel rhodopsin in the dorsal cochlear nucleus. These are widely utilized in studies of cortex and the potential problems revealed in this Ms therefore suggests caution in the interpretation of those studies. There are also interesting results re the physiology of VCN stellate cells to DCN.

There are, however, many technical issues that must be resolved before this ms is acceptable for publication in eNeuro. I have included the full reviews or two expert reviewers - they give a detailed breakdown of the corrections that must be made.

The reviewers also noted problems with the statistical methods. Statistics in Figure 3 E-F should be corrected for multiple comparisons, as ‘S’ and ‘S+L’ groups are tested in both E and F. Also, there should be statistical tests for differences between responding (R) and non-responding (NR) groups in Figures 4F and 7F.

With regard to the multimedia/extended data, the reviewers noted that the CLARITY video does not seem to be integral to the study. It does not contain additional insight into Chrna2 labeling that is not already present in Figure 6.

If the authors still want to include this video, they should enunciate what it will add to their manuscript.

Reviewer #1

The major conclusions of this paper are that:

1) Chr2/Arch viral expression driven by the CaMKIIa promoter (which in cortex, is generally assumed as specific to excitatory neurons) are not specific to the major output neurons of the DCN, fusiform/giant cells.

2) Activating or silencing DCN neurons via CaMKIIa driven expression of Chr2 or Arch causes bi-directional and counter-intuitive effects. These results are not surprising to me given the non-specificity of the viral method and known recurrence of the DCN circuit. Nevertheless, they provide a cautionary tale of how optogenetic manipulations, often assumed as having straightforward effects on circuit function, may yield very difficult to interpret results.

3) Chrna2-cre line labels neurons in the VCN, likely t-stellates and bushy cells. This conclusion is mainly based on fiber tracts labeled from Chrna2-cre x Ai14 crosses.

I think the most interesting result here is that the authors observed, albeit in a minority of cells, that activating Chrna2-cre neurons can have excitatory effects in the DCN. This is interesting because it’s been long hypothesized based on anatomical reconstructions that T-stellate cells provide excitatory inputs to the DCN, yet physiological evidence is lacking. Although it is difficult to establish from the experiments herein that the effects are due to a direct T-stellate->DCN projection, they nevertheless provide some supporting circumstantial clues.

From a conceptual level, the work is suitable for the journal. I do however have a number of concerns regarding methodology, data presentation, and analyses. I hope that the authors will find these comments helpful in strengthening the manuscript.

Major:

I cannot find anywhere in the manuscript if the authors tested the effect of light stimulation protocols in uninjected control mice. This is a pretty standard control for these kinds of experiments, and is all the more necessary given recent work from the Kreitzer lab showing that laser light can heat tissue and change neuronal physiology independent of opsin expression. Presumably, the authors will not observe any light-evoked excitation or shifts in response timing in uninjected controls.

Given the hypothesized non-specificity of the CamKII virus, could the authors comment on what type of units were directly light excited? Do the authors have FRAs they can show to determine if these are complex spiking, type II, or type III/IV, which we know from previous work map onto distinct cell-types? In absence of morphological reconstructions (e.g., cell fills from intracellular recordings) which are beyond the scope of this study, it is a bit loose to conclude that different cell classes are being labeled based on a few pictures showing "small" and "large" YFP-labeled somata. At the very least, some quantification of soma size and distribution in the DCN would provide more substantive evidence for this claim. For example, we know that the upper molecular layer contains mostly cell bodies of inhibitory cartwheel and stellate interneurons...are these cells labeled in their injected mice?

Figure 2:

This method of recording ABRs seems...unconventional. Typically ABR signals are not recorded from electrodes placed directly in the DCN. In addition, the Y-axis of the example traces (8-12 mV) and summary graph (75 mV!) seem at least one order of magnitude greater than what should be typically observed from field potentials or ABRs. I’m assuming this is a typo or some gain calibration is incorrect somewhere.

Furthermore, even if these are ABR signals with different peaks representing activity in separate central auditory stations, I would argue that it is formally difficult to conclude that these are "normal" ABRs from the data presented. Indeed, raw ABR amplitudes don’t mean a whole lot as they are highly dependent on electrode placement, and assessing how "normal" these values are would require a non-injected control group. Rather, if the authors presented sounds of different amplitudes in these experiments, they could quantify the "ABR" threshold and cross-reference those data to previous literature. However, it would be first necessary for the authors to provide a citation for their methodology of recording ABR responses from an electrode in the DCN. Finally, In the summary graph for the ABR peaks, it is unclear what the triangle, bars, and circles represent.

Minor:

Figure 3: Could the authors show example rasters for sound + light condition? Even better would be to show, for the same unit, sound, light, *and* sound + light conditions.

Figure 3E: Given that multiple significance tests seem to be run on the same dataset, a series of t-tests (uncorrected for multiple comparisons) is not the most appropriate here.

Figure 7B: the summary histogram looks like spiking increases before the sound onset. The authors might want to correct this and make sure this is simply a presentation/binning issue rather than anything fishy in their analysis pipeline. Same thing seems observable in far right panel of 7C with respect to light onset.

Figure 7F: The combination of colors, along with the small effect size, make it difficult to assess the claim that there is a change in firing rate with light stimulation. Consider changing the colors here?

there are a number of typos throughout the manuscript

-line 34, "receives" should be receive

-line 248 refers to Figure 8, but there is no Figure 8 in the manuscript

-Figure 7 legend, DNC should be DCN

...and so on

The authors may want to thoroughly proof-read prior to resubmission.

Discussion:

"Attempts to engineer viral vectors with population specific promoters have not produced useful tools for cell specificity."

This sentence should be toned down or removed. Many viral tools, while not perfectly cell-type specific, display enough cell-type biases to be "useful tools" for biological research.

Reviewer #2

In the manuscript "Using cortical neuron markers to target cells in the dorsal cochlear nucleus," the authors test the expression and function of the markers CaMKIIa and Chrna-2 in cell populations in the dorsal cochlear nucleus (DCN). The authors highlight that although such markers have been used extensively in neocortical areas, the application of these markers to brainstem areas such as the DCN is unclear. The authors attempt to fill this knowledge gap by testing the expression pattern of CaMKIIa in the DCN, as well demonstrating that activating/inhibiting these neurons causes diverse effects. They also demonstrate that in the Chrna2-cre mouse line, Chrna2 is not expressed in the DCN, but in the VCN, and activating this pathway modulates the DCN. Overall, this work advances the field by revealing that CaMKIIa does not label a distinct population of excitatory neurons in the DCN, and that manipulating this population has diverse effects on DCN sound processing. Moreover, the authors demonstrate that Chrna2 cells in the VCN increase DCN firing. Overall, this work contains interesting findings, but with a few major and minor concerns, outlined in detail below.

Major comments:

1. The authors state in Results section 3.1 that control viruses (e.g. not expressing ChR) were injected. Did the authors perform these controls for all experimental groups? If so, they are not stated in the Methods or shown in Figures. These would be informative controls for verifying that the effects of the light stimulation are indeed due to the expressed opsin. The authors could demonstrate that the light stimulation does not affect responses in the control injected group, in comparison to the opsin-expressing group.

2. The authors seem to over-interpret the histology results in Figure 2. While it indeed appears that CaMKIIa labels a diverse population, the statement that "the CaMKIIa promoter is not specific for DCN excitatory neurons" (line 295) cannot be definitively concluded based solely on morphological criteria. If possible, the authors should consider co-labeling the tissue with other excitatory/inhibitory markers (such as vGlut, GAD, GlyT2) to further characterize the CaMKIIa population(s). Alternatively, the authors should quantify the proportion of labeled neurons in distinct morphological categories, and expand discussion on the known morphological differences between fusiform cells, cartwheel cells, granule cells, etc.

3. It is not clear how targeting these neuron populations, and in particular the CaMKIIa population, would be "useful for manipulating the DCN" as the authors claim. On the contrary, the authors’ data suggest that CaMKIIa labels a heterogeneous population, with diverse and seemingly unpredictable effects upon activation (Fig. 3, some increase firing, some decrease firing). The data are still informative, but perhaps the authors should consider emphasizing the diverse results as a word of caution for other investigators using these markers in the DCN, rather than a potential translational intervention. Moreover, it is not clear what advantage these markers would have over other (already used) markers such as VGLut2, GlyT2, and VGAT.

Minor comments:

1. In the Introduction, the section about PV and somatostatin expression (lines 60-66) can be shortened or removed. It is not clear how this topic is relevant to the current work, as these markers are not studied.

2. Figure 2 A-C: the images should include scale bars

3. Methods 2.8: ABRs: the authors should describe in more detail how ABRs were extracted from extracellular unit recordings

4. How do the authors explain the decrease in firing rate observed in the sound+light condition (Figure 3)? Some additional discussion on this unexpected result would be appreciated.

5. Statistics in Figure 3 E-F should be corrected for multiple comparisons, as ‘S’ and ‘S+L’ groups are tested in both E and F. Also, there should be statistical tests for differences between responding (R) and non-responding (NR) groups in Figures 4F and 7F.

6. Figure 6 legend: scale bar lengths are stated for A, B, C, but not D-F

7. Figure 7 B: this example unit looks like it has a much higher baseline firing rate than the other units shown (perhaps the firing rate is so high it is masking changes due to sound/light). The authors should consider showing a more representative neuron.

8. It is interesting that Chrna2 (used to label interneurons in cortex) labels putative T-stellate cells in VCN. The authors should further discuss this unexpected result.

9. Discussion lines 445-446: "based on literature" - should cite references here

10. In the graphical abstract, for ‘CaMKIIa-ChR2’ the purple arrow next to ‘Sound+Light’ seems to indicate increased firing rate, but the results actually show that sound+light decreased firing rate compared to sound alone (Figure 3E-F). For ‘DIO-ChR2’ (assumed to be from Figure 7D), the left plot should be ‘Light’ and the right plot ‘Sound+Light’.

11. The multimedia/extended data (CLARITY video) does not seem to be integral to the study. It does not contain additional insight into Chrna2 labeling that is not already present in Figure 6.

12. Minor typos:

Line 34: "receive"

Line 160: "split"

Line 209: "depicted"

Line 269: "described"

Line 344: "responses"

Line 365: "respond to sound stimulation"

Line 446: "patterns"

## Author Response

Response to Review:

Manuscript: Using cortical neuron markers to target cells in the dorsal cochlear nucleus

Comments on the Visual Abstract for Author (Required):

In the graphical abstract, for ‘CaMKIIa-ChR2’ the purple arrow next to ‘Sound+Light’ seems to indicate increased firing rate, but the results actually show that sound+light decreased firing rate compared to sound alone (Figure 3E-F). For ‘DIO-ChR2’ (assumed to be from Figure 7D), the left plot should be ‘Light’ and the right plot ‘Sound+Light’.

Purple arrow and the ‘Light’ label were corrected. Some additional minor changes were made to improve the final image quality of the Graphical abstract.

Editorial comments

The manuscript provides a useful technical advance by testing the effects of expression of

CaMKIIa and channelrhodopsin in the dorsal cochlear nucleus. These are widely utilized in studies of cortex and the potential problems revealed in this Ms therefore suggests caution in the interpretation of those studies. There are also interesting results re the physiology of VCN stellate cells to DCN.

There are, however, many technical issues that must be resolved before this ms is acceptable for publication in eNeuro. I have included the full reviews or two expert reviewers - they give a detailed breakdown of the corrections that must be made.

The reviewers also noted problems with the statistical methods.

Statistics in Figure 3 E-F should be corrected for multiple comparisons, as ‘S’ and ‘S+L’ groups are tested in both E and F.

Indeed both the ‘S’ and ‘S+L’ datasets are compared twice. P-values were bonferroni-adjusted for multiple comparisons and a statement was added in Methods/Unit analysis.

Also, there should be statistical tests for differences between responding (R) and non-responding

(NR) groups in Figures 4F and 7F.

Statistical test have been applied for both panels as described in Methods/Unit Analysis section, and the p values are now reported in the respective figure legends.

Reviewer #1

The major conclusions of this paper are that:

A) Chr2/Arch viral expression driven by the CaMKIIa promoter (which in cortex, is generally assumed as specific to excitatory neurons) are not specific to the major output neurons of the

DCN, fusiform/giant cells.

B) Activating or silencing DCN neurons via CaMKIIa driven expression of Chr2 or Arch causes bidirectional and counter-intuitive effects. These results are not surprising to me given the nonspecificity of the viral method and known recurrence of the DCN circuit. Nevertheless, they provide a cautionary tale of how optogenetic manipulations, often assumed as having straightforward effects on circuit function, may yield very difficult to interpret results.

2

C) Chrna2-cre line labels neurons in the VCN, likely t-stellates and bushy cells. This conclusion is mainly based on fiber tracts labeled from Chrna2-cre x Ai14 crosses.

I think the most interesting result here is that the authors observed, albeit in a minority of cells, that activating Chrna2-cre neurons can have excitatory effects in the DCN. This is interesting because it’s been long hypothesized based on anatomical reconstructions that T-stellate cells provide excitatory inputs to the DCN, yet physiological evidence is lacking. Although it is difficult to establish from the experiments herein that the effects are due to a direct T-stellate->DCN projection, they nevertheless provide some supporting circumstantial clues.

From a conceptual level, the work is suitable for the journal. I do however have a number of concerns regarding methodology, data presentation, and analyses. I hope that the authors will find these comments helpful in strengthening the manuscript.

Major:

1) I cannot find anywhere in the manuscript if the authors tested the effect of light stimulation protocols in uninjected control mice. This is a pretty standard control for these kinds of experiments, and is all the more necessary given recent work from the Kreitzer lab showing that laser light can heat tissue and change neuronal physiology independent of opsin expression.

Presumably, the authors will not observe any light-evoked excitation or shifts in response timing in uninjected controls.

We agree and have now included control experiments from uninjected animals. As we use 5- 7mW/mm2 for both blue and green laser output we intend to not produce heat induced currents that could affect our results. Our control experiments show no significant changes in overall firing rate or change in number of spikes in response to sound pulses when light stimulation was provided to non-injected animals (shown in the new additional Figure 8A-C). This is in agreement with Owen et al (2019 - Kreitzer lab) that showed that 15mW/mm2 green (532nm) light can alter firing rate of certain neurons, but 3mW/mm2 had little effect on firing, although both 15mW/mm2 and 3mW/mm2 light stimulation induced small light-induced currents (∼18 and 1-2pA, respectively) due to heating.

2a) Given the hypothesized non-specificity of the CamKII virus, could the authors comment on what type of units were directly light excited? Do the authors have FRAs they can show to determine if these are complex spiking, type II, or type III/IV, which we know from previous work map onto distinct cell-types? In absence of morphological reconstructions (e.g., cell fills from intracellular recordings) which are beyond the scope of this study, it is a bit loose to conclude that different cell classes are being labeled based on a few pictures showing "small" and "large" YFPlabeled somata.

At the very least, some quantification of soma size and distribution in the DCN would provide more substantive evidence for this claim. For example, we know that the upper molecular layer contains mostly cell bodies of inhibitory cartwheel and stellate interneurons...are these cells labeled in their injected mice?

Yes, frequency response area (FRA), or sensory (spectral) receptive fields, can classify distinct cell-types in auditory areas (for ex. Palmer et al., 2013) but was not analyzed here as we did not

3 use pure tones at different intensities, we used noise (5-15kHz) at a single intensity (80dBSPL) which is not optimal for producing FRAs (added to Discussion at line 547-554). Furthermore, the thickness of slices prevents us from correlating cell morphology with specific units recorded. Also the spread of injected virus makes cell labeling not uniform across the DCN hampering thorough histology.

However, we took great care in correlating unit responses with the probe depth and now added depth profile plots of all recorded units (Figure 8D-E). Units responding to light are color-coded (blue) showing that units responding to light are spread across the DCN DV axis, which supports the conclusion that different cell types are being recorded (due to the layered arrangement of cells in the DCN). We cannot say if stellate interneurons are labelled, but we believe some inhibitory cartwheel cells were labeled as we saw small round somata labeled in the vicinity of larger fusiform cells, and we recorded dis-inhibition like responses, indicating some cells activated by light were inhibitory. Whole-cell patch clamp recordings of eYFP+ cells could for example investigate complex-spiking activity, but this was not the scope of this study.

Figure 8 is added to the Results and Discussion, and depth coordinates are now shown in Figure 3

B-D instead of unit number.

3) Figure 2:

This method of recording ABRs seems...unconventional. Typically ABR signals are not recorded from electrodes placed directly in the DCN. In addition, the Y-axis of the example traces (8-12 mV) and summary graph (75 mV!) seem at least one order of magnitude greater than what should be typically observed from field potentials or ABRs. I’m assuming this is a typo or some gain calibration is incorrect somewhere.

Furthermore, even if these are ABR signals with different peaks representing activity in separate central auditory stations, I would argue that it is formally difficult to conclude that these are "normal" ABRs from the data presented. Indeed, raw ABR amplitudes don’t mean a whole lot as they are highly dependent on electrode placement, and assessing how "normal" these values are would require a non-injected control group. Rather, if the authors presented sounds of different amplitudes in these experiments, they could quantify the "ABR" threshold and cross-reference those data to previous literature. However, it would be first necessary for the authors to provide a citation for their methodology of recording ABR responses from an electrode in the DCN. Finally, In the summary graph for the ABR peaks, it is unclear what the triangle, bars, and circles represent.

This method of recording ABRs is indeed unconventional, since, if the aim of the experiment was to solely record ABRs, there would be no need for intracerebral electrodes. However, since our experiments involved recording neural signals during auditory stimulation, we showed that both extracellular spikes and ABR waves (a high-frequency event-related LFP) can be extracted. We added the following sentence to the ‘Methods/Auditory brainstem responses’ section with citations to the methodology:

"Auditory brainstem responses (ABRs) are high-frequency event-related potentials that are commonly recorded from surface electrodes placed over the skin of the head or subdermally over the skull, but can also be recorded from auditory central areas (Lev and Sohmer, 1972; Achor and

Starr, 1980), or even from brain areas unrelated to auditory processing (Jewett, 1970)."

The typo of the axis unit was corrected in Fig 2D and E (mV -> μV).

4

We agree that we cannot assume hearing normality based on the experimental data. We can only claim that neither the viral vector injection procedure nor the concomitant light stimulation prevented the animals from hearing the provided sound stimulus, since all animals showed at least

5 peaks in the ABR wave in response to sound.

We made the following changes:

● Changed the title of the ‘Result section’ from "Auditory brainstem responses are normal during optogenetic excitation of CaMKIIα-ChR2 positive DCN neurons" to "Auditory brainstem responses can be extracted during optogenetic excitation of CaMKIIα-ChR2 positive DCN neurons";

● Removed "normal" from Figure 2 legend title;

● Changed the first sentence of the same section from "To confirm that viral constructs do not cause any abnormalities in hearing we routinely [...]" to "To confirm that animals can hear the provided sound stimulation after injection of viral constructs we routinely [...]";

-Changed the sentence "We found all animals to display normal mean ABR waveforms [...]" to

"We found all animals to display at least five peaks in the ABR waveforms [...]";

-Changed the sentence "Together this show that the animal’s hearing at the stimulus intensity was not impaired by the viral vector injection procedure or by concomitant blue light stimulation." to

"Together this shows that the animals can hear the provided sound stimulus after viral vector injection procedure and during concomitant blue light stimulation.".

Furthermore, clarification of bars, triangles and circles from Figure 2D-E, was added to the Fig2 legend: "Center, box plots show group amplitude of the first 5 ABR peaks, horizontal lines show the median, triangles show mean, circles show outliers, whiskers bounding 99% of the data points.

Right, group latency of the first 5 ABR peaks."

Minor:

1) Figure 3: Could the authors show example rasters for sound + light condition? Even better would be to show, for the same unit, sound, light, *and* sound + light conditions.

Yes, we agree. Units in Figure 3C and D now show the Sound, Light and Sound+Light conditions, and the figure legend was updated accordingly.

2) Figure 3E: Given that multiple significance tests seem to be run on the same dataset, a series of t-tests (uncorrected for multiple comparisons) is not the most appropriate here.

P-values were bonferroni-corrected for multiple comparisons and description was added to

Methods/Unit Analysis.

3) Figure 7B: the summary histogram looks like spiking increases before the sound onset. The authors might want to correct this and make sure this is simply a presentation/binning issue rather

5 than anything fishy in their analysis pipeline. Same thing seems observable in far right panel of 7C with respect to light onset.

Figure 7B was indeed a binning issue. We decreased the bin size from 2ms to 1ms and the

"increase" in spiking activity before the sound pulse disappeared.

This comment also highlighted that the PSTH bin size was written as 1ms (this typo was now corrected to 2ms in the Methods/Unit analysis section - with the exception for the legend of Figure 7B).

Figure 7C had a different issue. The alignment of responses was for the Sound stimulation, therefore, the light pulse should be from -4 to 6ms while the sound pulse should be from 0 to 3ms.

We corrected the stimulation indication position in the PSTH and Raster plots of Sound+Light stimulation.

4) Figure 7F: The combination of colors, along with the small effect size, make it difficult to assess the claim that there is a change in firing rate with light stimulation. Consider changing the colors here?

Changed baseline data points to black.

5) there are a number of typos throughout the manuscript

-line 34, "receives" should be receive

-line 248 refers to Figure 8, but there is no Figure 8 in the manuscript

-Figure 7 legend, DNC should be DCN ...and so on

The authors may want to thoroughly proof-read prior to resubmission.

Corrected and proof-read for additional grammatical errors and typos.

6) Discussion:

"Attempts to engineer viral vectors with population specific promoters have not produced useful tools for cell specificity."

This sentence should be toned down or removed. Many viral tools, while not perfectly cell-type specific, display enough cell-type biases to be "useful tools" for biological research.

Agreed and the sentence have been changed from:

"There is a large variety of genetic tools for dissecting the role of specific neuronal populations.

The vast majority of these tools are transgenic mice expressing a reporter protein or a recombinase. Attempts to engineer viral vectors with population specific promoters have not produced useful tools for cell specificity." To

"There is a large variety of genetic tools for dissecting the role of specific neuronal populations, such as transgenic mice expressing a reporter protein or a recombinase, or viral vectors with different promoters for certain cell specificity".

6

Reviewer #2

In the manuscript "Using cortical neuron markers to target cells in the dorsal cochlear nucleus," the authors test the expression and function of the markers CaMKIIa and Chrna-2 in cell populations in the dorsal cochlear nucleus (DCN). The authors highlight that although such markers have been used extensively in neocortical areas, the application of these markers to brainstem areas such as the DCN is unclear. The authors attempt to fill this knowledge gap by testing the expression pattern of CaMKIIa in the DCN, as well demonstrating that activating/inhibiting these neurons causes diverse effects. They also demonstrate that in the Chrna2-cre mouse line, Chrna2 is not expressed in the DCN, but in the VCN, and activating this pathway modulates the DCN. Overall, this work advances the field by revealing that CaMKIIa does not label a distinct population of excitatory neurons in the DCN, and that manipulating this population has diverse effects on DCN sound processing. Moreover, the authors demonstrate that Chrna2 cells in the VCN increase DCN firing.

Overall, this work contains interesting findings, but with a few major and minor concerns, outlined in detail below.

Major comments:

1. The authors state in Results section 3.1 that control viruses (e.g. not expressing ChR) were injected. Did the authors perform these controls for all experimental groups? If so, they are not stated in the Methods or shown in Figures. These would be informative controls for verifying that the effects of the light stimulation are indeed due to the expressed opsin. The authors could demonstrate that the light stimulation does not affect responses in the control injected group, in comparison to the opsin-expressing group.

We agree that this is an important point. Here, the animals injected with the control vector were not recorded, they were used initially for validation of expression pattern. We have now added data recorded from non-injected animals, and made an additional Figure 8A-C, showing that blue light do not evoke responses in control animals and that 20s green light do not alter or delay the sound responses in control animals due to any minor heating of the tissue (Owen et al., 2019) at the light intensity used in this work (5-7mW/mm2

). Figure 8 is added to Results and Discussion.

2. The authors seem to over-interpret the histology results in Figure 2. While it indeed appears that

CaMKIIa labels a diverse population, the statement that "the CaMKIIa promoter is not specific for

DCN excitatory neurons" (line 295) cannot be definitively concluded based solely on morphological criteria. If possible, the authors should consider co-labeling the tissue with other excitatory/inhibitory markers (such as vGlut, GAD, GlyT2) to further characterize the CaMKIIa population(s). Alternatively, the authors should quantify the proportion of labeled neurons in distinct morphological categories, and expand discussion on the known morphological differences between fusiform cells, cartwheel cells, granule cells, etc.

We have clarified the results such as, in comparison with the neocortex and hippocampus, the "[...]

CaMKIIα promoter is not specific for the DCN excitatory fusiform cell layer, [...]" (line 298). We have also included depth profile plots in Figure 8D-E to show that light responding units are spread along the DCN DV axis, supporting the claim that different cell types are expressing the opsin. Also

Figure 3 B-D now shows depth coordinates of the DCN units instead of unit number along the probe shank. A thorough morphological study using excitatory and inhibitory markers would indeed be interesting but was not the scope of this study, as we here aimed to investigate two different markers for controlling neuronal activity in the DCN in response to sound.

7

A sentence was also modified in the Discussion (original line 457):

"In this work, we first show that the CaMKIIα promoter targets DCN neurons of different morphology, and at different depths of the DCN, and thereby not only putative fusiform cells/fusiform cell layer."

3. It is not clear how targeting these neuron populations, and in particular the CaMKIIa population, would be "useful for manipulating the DCN" as the authors claim. On the contrary, the authors’ data suggest that CaMKIIa labels a heterogeneous population, with diverse and seemingly unpredictable effects upon activation (Fig. 3, some increase firing, some decrease firing). The data are still informative, but perhaps the authors should consider emphasizing the diverse results as a word of caution for other investigators using these markers in the DCN, rather than a potential translational intervention. Moreover, it is not clear what advantage these markers would have over other (already used) markers such as VGLut2, GlyT2, and VGAT.

We agree that we have been unclear with what we mean by ‘useful for manipulating’. We found that the CaMKIIa promoter can be used to optogenetically lower or scramble timing of neuronal activity of the DCN but not to specifically activate the DCN in a temporally precise manner or increase response to sound. As the DCN is implicated in tinnitus, the CaMKIIa promoter could provide a useful, low-cost (compared to transgenic animals) option for lowering overall DCN activity if used in combination with chemogenetics, for example.

We have modified the Conclusion to fit this line of reasoning.

We are also grateful for this comment as it highlighted a confusing citation. In Discussion the sentence: "In slice preparations, vesicular glutamate transporter 2 (VGluT2) transgenic mice have already been used for targeting and controlling DCN fusiform cell firing (Apostolides and Trussell, 2013a)." have been corrected in line 498:

"In slice preparations, vesicular glutamate transporter 2 (VGluT2) transgenic mice have already been used for targeting DCN fusiform and stellate cells to investigate control of DCN fusiform cell activity (Apostolides and Trussell, 2013a).

Thereby VGlut2 transgenic animals can not be used to specifically target fusiform cells.

Minor comments:

1. In the Introduction, the section about PV and somatostatin expression (lines 60-66) can be shortened or removed. It is not clear how this topic is relevant to the current work, as these markers are not studied.

We agree. Original lines 60-66 have been shortened to (line 60-63):

"Cortical interneuron markers could also be used to tag DCN cells. However, for two commonly used markers, both parvalbumin and somatostatin expression can be quite promiscuous in the neocortex/hippocampus region (Kawaguchi and Kondo, 2002; Mikulovic et al., 2015) and in the

DCN, parvalbumin is distributed across layers without apparent population specificity and somatostatin shows weak expression (Lein et al., 2007). Nonetheless, recently [...]"

8

2. Figure 2 A-C: the images should include scale bars

Scale bars were added and described in the figure legend.

3. Methods 2.8: ABRs: the authors should describe in more detail how ABRs were extracted from extracellular unit recordings

We have added the following section to the ABR methods:

"Auditory brainstem responses (ABRs) are high-frequency event-related potentials that are commonly recorded from surface electrodes placed over the skin of the head or subdermally over the skull, but can also be recorded from auditory central areas (Lev and Sohmer, 1972; Achor and

Starr, 1980), or even from brain areas unrelated to auditory processing (Jewett, 1970)."

And modified to next part to:

"Here, ABRs were extracted from the intracerebral electrode for recording extracellular spikes in the DCN. The high-frequency event-related local field potential was filtered (4th order butterworth digital bandpass filter from 500 to 1500Hz), sliced (3ms before and 12ms after each sound pulse) and averaged, and ABR peaks were detected [...]"

4. How do the authors explain the decrease in firing rate observed in the sound+light condition (Figure 3)? Some additional discussion on this unexpected result would be appreciated.

A paragraph was expanded (previous lines 522-526) in Discussion providing possible explanations for the observed decrease in number of spikes elicited by sound+light compared to sound alone

(line 554-565):

"One of the interesting findings of this study was that sound pulses alone elicited more spikes than sound+light stimuli. There are different possible explanations for this, such as inhibitory neurons express opsins and thus the observed decrease could be part of motifs of feed-forward inhibition

(Roberts and Trussell, 2010). Or, the fact that the light stimulus is slightly longer than the sound pulse, thereby the light pulse could immediately activate inhibitory neurons just before the sound pulse, which would decrease the probability of the circuitry to be activated compared to the sound pulse alone. Another possible explanation, for excitatory neurons expressing ChR2, is that the unit would be activated by the light pulse, and next be in a non-excitable state when the sound pulse is presented. This "light-on-sound" masking was shown before (Hernandez et al, 2014), where expressing ChR2 in cochlear spiral ganglion neurons and presenting the sound stimulus embedded in blue light stimulation masked the evoked auditory brainstem response, although not completely abolished (Hernandez et al., 2014)."

5. Statistics in Figure 3 E-F should be corrected for multiple comparisons, as ‘S’ and ‘S+L’ groups are tested in both E and F. Also, there should be statistical tests for differences between responding (R) and non-responding (NR) groups in Figures 4F and 7F.

Agreed. Both ‘S’ and ‘S+L’ datasets were compared twice, and p-values were bonferroni-adjusted for multiple comparisons and a statement was added in Methods/Unit analysis. Also the missing

9

statistical tests were performed as described in Methods/Unit Analysis and the resulting p values were added to the respective figure legends.

6. Figure 6 legend: scale bar lengths are stated for A, B, C, but not D-F

This was a typo in the legend. The statement was changed from

"Scale bars: 1mm (A) and 500μm (B and C)"

to

"Scale bars: 1mm (A) and 500μm (B-F)."

7. Figure 7 B: this example unit looks like it has a much higher baseline firing rate than the other units shown (perhaps the firing rate is so high it is masking changes due to sound/light). The authors should consider showing a more representative neuron.

As reported in the section "Results/VCN Chrna2 positive neurons can generate indirect optogenetic activation of DCN neurons", unfortunately only 2 units responded only to sound stimulation, and both have firing rate >30Hz. However, to improve the visualization of the response

we decreased the bin size from 2ms to 1ms and decreased the line thickness for raster ticks and the PSTH average line.

8. It is interesting that Chrna2 (used to label interneurons in cortex) labels putative T-stellate cells in VCN. The authors should further discuss this unexpected result.

This section has been added to Discussion (previous line 528) now line 567-581:

"T-stellate cells, also called type I/multipolar/choppers, are excitatory neurons of the VCN with diverse connectivity but project mainly to the contralateral inferior colliculus (reviewed by Oertel et al., 2011). T-stellate cells have been shown to have axon collaterals that go into the deep layers of

DCN without reaching the fusiform cell layer (Oertel et al., 2011) which is similar to the diffuse axon terminals we observed (Figure 7A, and CLARITY data). Our virus labeling also corroborates the indication that T-stellate cells provide input to the ipsilateral LSO, as we observed strong labeling of the ipsilateral LSO. However, bushy cells of the AVCN also project to the ipsilateral LSO, making further experiments needed to confirm the degree of such connectivity. It is also known that

T-stellate cells are modulated by nicotinergic agonists while D-stellate cells are not (Fujino & Oertel 2001), in agreement with our cre-line that specifically targets neurons with nicotinic receptors containing the nicotinic acetylcholine receptor subunit 2 alpha. Recently, T-stellate cells were shown to innervate neighboring T-stellate cells responding to similar sound frequencies, regulating local excitation through nitric oxide-dependent plasticity mechanisms (Cao et al., 2019). The

Chrna2-cre line could possibly aid in studies of hyperexcitability in T-stellate cells relating to hearing loss and/or tinnitus (Cao et al., 2019; Coomber et al., 2014). Here, using Chrna2-cre mice and [...]"

9. Discussion lines 445-446: "based on literature" - should cite references here

Corrected (line 466):

"[...] based on literature and projection patterns" changed to "[...] based on pharmacology and projection patterns (Oertel et al., 2011)".

10

10. In the graphical abstract, for ‘CaMKIIa-ChR2’ the purple arrow next to ‘Sound+Light’ seems to indicate increased firing rate, but the results actually show that sound+light decreased firing rate compared to sound alone (Figure 3E-F). For ‘DIO-ChR2’ (assumed to be from Figure 7D), the left plot should be ‘Light’ and the right plot ‘Sound+Light’.

Purple arrow and ‘Light’ label corrected.

11. The multimedia/extended data (CLARITY video) does not seem to be integral to the study. It does not contain additional insight into Chrna2 labeling that is not already present in Figure 6.

12. Minor typos:

Line 34: "receive"

Line 160: "split"

Line 209: "depicted"

Line 269: "described"

Line 344: "responses"

Line 365: "respond to sound stimulation"

Line 446: "patterns"

Corrected and proof-read for additional grammatical errors and typos.

## References

[B1] Achor L, Starr A (1980) Auditory brain stem responses in the cat. i. intracranial and extracranial recordings. Electroencephalogr Clin Neurophysiol 48:154–173. 10.1016/0013-4694(80)90301-6 6153332

[B2] Apostolides PF, Trussell LO (2013a) Rapid, activity-independent turnover of vesicular transmitter content at a mixed glycine/GABA synapse. J Neurosci 33:4768–4781. 10.1523/JNEUROSCI.5555-12.2013 23486948 PMC3639006

[B3] Apostolides PF, Trussell LO (2013b) Regulation of interneuron excitability by gap junction coupling with principal cells. Nat Neurosci 16:1764–1772. 10.1038/nn.3569 24185427 PMC3963432

[B4] Ash C, Dubec M, Donne K, Bashford T (2017) Effect of wavelength and beam width on penetration in light-tissue interaction using computational methods. Lasers Med Sci 32:1909–1918. 10.1007/s10103-017-2317-4 28900751 PMC5653719

[B5] Baizer JS, Manohar S, Paolone NA, Weinstock N, Salvi RJ (2012) Understanding tinnitus: the dorsal cochlear nucleus, organization and plasticity. Brain Res 1485:40–53. 10.1016/j.brainres.2012.03.044 22513100 PMC3402636

[B6] Boyden ES, Zhang F, Bamberg E, Nagel G, Deisseroth K (2005) Millisecond-timescale, genetically targeted optical control of neural activity. Nat Neurosci 8:1263–1268. 10.1038/nn1525 16116447

[B7] Cao XJ, Lin L, Sugden AU, Connors BW, Oertel D (2019) Nitric oxide-mediated plasticity of interconnections between t-stellate cells of the ventral cochlear nucleus generate positive feedback and constitute a central gain control in the auditory system. J Neurosci 39:6095–6107. 10.1523/JNEUROSCI.0177-19.2019 31160538 PMC6668202

[B8] Caswell TA, Droettboom M, Hunter J, Firing E, Lee A, Klymak J, Stansby D, Sales de Andrade E, Hedegaard Nielsen J, Varoquaux N, Root B, Hoffmann T, Elson P, May R, Dale D, Lee JJ, Seppänen JK, McDougall D, Straw A, Hobson P, et al. (2019) matplotlib/matplotlib v2.2.4. Available from https://zenodo.org/record/2669103#.YBmaZOhKg2w. Zenodo.

[B9] Chow BY, Han X, Dobry AS, Qian X, Chuong AS, Li M, Henninger MA, Belfort GM, Lin Y, Monahan PE, Boyden ES (2010) High-performance genetically targetable optical neural silencing by light-driven proton pumps. Nature 463:98–102. 10.1038/nature08652 20054397 PMC2939492

[B10] Coomber B, Berger JI, Kowalkowski VL, Shackleton TM, Palmer AR, Wallace MN (2014) Neural changes accompanying tinnitus following unilateral acoustic trauma in the guinea pig. Eur J Neurosci 40:2427–2441. 10.1111/ejn.12580 24702651 PMC4215599

[B11] Coomber B, Kowalkowski VL, Berger JI, Palmer AR, Wallace MN (2015) Modulating central gain in tinnitus: changes in nitric oxide synthase in the ventral cochlear nucleus. Front Neurol 6:53. 10.3389/fneur.2015.00053 25806021 PMC4354362

[B12] Darrow KN, Slama MCC, Kozin ED, Owoc M, Hancock K, Kempfle J, Edge A, Lacour S, Boyden E, Polley D, Brown MC, Lee DJ (2015) Optogenetic stimulation of the cochlear nucleus using channelrhodopsin-2 evokes activity in the central auditory pathways. Brain Res 1599:44–56. 10.1016/j.brainres.2014.11.044 25481416 PMC4859340

[B13] Davis RR, Newlander J, Ling X-B, Cortopassi GA, Krieg EF, Erway LC (2001) Genetic basis for susceptibility to noise-induced hearing loss in mice. Hear Res 155:82–90. 10.1016/S0378-5955(01)00250-7 11335078

[B14] Dehmel S, Pradhan S, Koehler S, Bledsoe S, Shore S (2012) Noise overexposure alters long-term somatosensory-auditory processing in the dorsal cochlear nucleus–possible basis for tinnitus-related hyperactivity? J Neurosci 32:1660–1671. 10.1523/JNEUROSCI.4608-11.2012 22302808 PMC3567464

[B15] Devor A (2000) Is the cerebellum like cerebellar-like structures? Brain Res Brain Res Rev 34:149–156. 10.1016/s0165-0173(00)00045-x 11113505

[B16] Finlayson PG, Kaltenbach JA (2009) Alterations in the spontaneous discharge patterns of single units in the dorsal cochlear nucleus following intense sound exposure. Hear Res 256:104–117. 10.1016/j.heares.2009.07.006 19622390 PMC2778575

[B17] Franklin K, Paxinos G (2008) The mouse brain in stereotaxic coordinates. San Diego: Academic Press.

[B18] Fujino K, Oertel D (2001) Cholinergic modulation of stellate cells in the mammalian ventral cochlear nucleus. J Neurosci 21:7372–7383. 10.1523/JNEUROSCI.21-18-07372.2001 11549747 PMC6763002

[B19] Grossan M, Peterson DC (2017) Tinnitus. Treasure Island: StatPearls Publishing. 28613560

[B20] Han BI, Lee HW, Kim TY, Lim JS, Shin KS (2009) Tinnitus: characteristics, causes, mechanisms, and treatments. J Clin Neurol 5:11–19. 10.3988/jcn.2009.5.1.11 19513328 PMC2686891

[B21] Henry KR (1979) Auditory brainstem volume-conducted responses: origins in the laboratory mouse. J Am Aud Soc 4:173–178. 511644

[B22] Hernandez VH, Gehrt A, Reuter K, Jing Z, Jeschke M, Mendoza Schulz A, Hoch G, Bartels M, Vogt G, Garnham CW, Yawo H, Fukazawa Y, Augustine GJ, Bamberg E, Kügler S, Salditt T, de Hoz L, Strenzke N, Moser T (2014) Optogenetic stimulation of the auditory pathway. J Clin Invest 124:1114–1129. 10.1172/JCI69050 24509078 PMC3934189

[B23] Hight AE, Kozin ED, Darrow K, Lehmann A, Boyden E, Brown MC, Lee DJ (2015) Superior temporal resolution of chronos versus channelrhodopsin-2 in an optogenetic model of the auditory brainstem implant. Hear Res 322:235–241. 10.1016/j.heares.2015.01.004 25598479 PMC4465525

[B24] Hilscher MM, Leão RN, Edwards SJ, Leão KE, Kullander K (2017) Chrna2-martinotti cells synchronize layer 5 type a pyramidal cells via rebound excitation. PLOS Biol 15:e2001392. 10.1371/journal.pbio.2001392 28182735 PMC5300109

[B25] Hunter JD (2007) Matplotlib: a 2D graphics environment. Comput Sci Eng 9:90–95. 10.1109/MCSE.2007.55

[B26] Jewett DL (1970) Volume-conducted potentials in response to auditory stimuli as detected by averaging in the cat. Electroencephalogr Clin Neurophysiol 28:609–618. 10.1016/0013-4694(70)90203-8 4192837

[B27] Jones E, Oliphant T, Peterson P (2001) SciPy: open source scientific tools for Python. Available from http://www.scipy.org

[B28] Kaltenbach JA, Zhang J (2007) Intense sound-induced plasticity in the dorsal cochlear nucleus of rats: evidence for cholinergic receptor upregulation. Hear Res 226:232–243. 10.1016/j.heares.2006.07.001 16914276

[B29] Kaltenbach JA, Zhang J, Finlayson P (2005) Tinnitus as a plastic phenomenon and its possible neural underpinnings in the dorsal cochlear nucleus. Hear Res 206:200–226. 10.1016/j.heares.2005.02.013 16081009

[B30] Kawaguchi Y, Kondo S (2002) Parvalbumin, somatostatin and cholecystokinin as chemical markers for specific gabaergic interneuron types in the rat frontal cortex. J Neurocytol 31:277–287. 12815247 10.1023/a:1024126110356

[B31] Koehler SD, Pradhan S, Manis PB, Shore SE (2011) Somatosensory inputs modify auditory spike timing in dorsal cochlear nucleus principal cells. Eur J Neurosci 33:409–420. 10.1111/j.1460-9568.2010.07547.x 21198989 PMC3059071

[B32] Kraus K, Ding D, Jiang H, Lobarinas E, Sun W, Salvi R (2011) Relationship between noise-induced hearing-loss, persistent tinnitus and growth-associated protein-43 expression in the rat cochlear nucleus: does synaptic plasticity in ventral cochlear nucleus suppress tinnitus? Neuroscience 194:309–325. 10.1016/j.neuroscience.2011.07.056 21821100 PMC3390756

[B33] Leão RN, Mikulovic S, Leão KE, Munguba H, Gezelius H, Enjin A, Patra K, Eriksson A, Loew LM, Tort ABL, Kullander K (2012) OLM interneurons differentially modulate CA3 and entorhinal inputs to hippocampal CA1 neurons. Nat Neurosci 15:1524–1530. 10.1038/nn.3235 23042082 PMC3483451

[B34] Lein ES, Hawrylycz MJ, Ao N, Ayres M, Bensinger A, Bernard A, Boe AF, Boguski MS, Brockway KS, Byrnes EJ, Chen L, Chen L, Chen TM, Chin MC, Chong J, Crook BE, Czaplinska A, Dang CN, Datta S, Dee NR, et al. (2007) Genome-wide atlas of gene expression in the adult mouse brain. Nature 445:168–176. 10.1038/nature05453 17151600

[B35] Lev A, Sohmer H (1972) Sources of averaged neural responses recorded in animal and human subjects during cochlear audiometry (electro-cochleogram). Arch Klin Exp Ohren Nasen Kehlkopfheilkd 201:79–90. 10.1007/BF00341066 5051284

[B36] Levine RA (1999) Somatic (craniocervical) tinnitus and the dorsal cochlear nucleus hypothesis. Am J Otolaryngol 20:351–362. 10.1016/S0196-0709(99)90074-1 10609479

[B37] Lu HW, Trussell LO (2016) Spontaneous activity defines effective convergence ratios in an inhibitory circuit. J Neurosci 36:3268–3280. 10.1523/JNEUROSCI.3499-15.2016 26985036 PMC4792938

[B38] Lyngholm D, Sakata S (2019) Cre-dependent optogenetic transgenic mice without early age-related hearing loss. Front Aging Neurosci 11:29. 10.3389/fnagi.2019.00029 30863301 PMC6399395

[B39] Mahn M, Prigge M, Ron S, Levy R, Yizhar O (2016) Biophysical constraints of optogenetic inhibition at presynaptic terminals. Nat Neurosci 19:554–556. 10.1038/nn.4266 26950004 PMC4926958

[B40] Manzoor NF, Licari FG, Klapchar M, Elkin RL, Gao Y, Chen G, Kaltenbach JA (2012) Noise-induced hyperactivity in the inferior colliculus: its relationship with hyperactivity in the dorsal cochlear nucleus. J Neurophysiol 108:976–988. 10.1152/jn.00833.2011 22552192 PMC3424082

[B41] Mikulovic S, Restrepo CE, Hilscher MM, Kullander K, LeÃ£o RN (2015) Novel markers for OLM interneurons in the hippocampus. Front Cell Neurosci 9:201. 10.3389/fncel.2015.00201 26082683 PMC4451365

[B42] Mikulovic S, Restrepo CE, Siwani S, Bauer P, Pupe S, Tort ABL, Kullander K, Leão RN (2018) Ventral hippocampal OLM cells control type 2 theta oscillations and response to predator odor. Nat Commun 9:3638. 10.1038/s41467-018-05907-w 30194386 PMC6128904

[B43] Milinkeviciute G, Muniak MA, Ryugo DK (2017) Descending projections from the inferior colliculus to the dorsal cochlear nucleus are excitatory. J Comp Neurol 525:773–793. 10.1002/cne.24095 27513294

[B44] Noben-Trauth K, Zheng QY, Johnson KR (2003) Association of cadherin 23 with polygenic inheritance and genetic modification of sensorineural hearing loss. Nat Genet 35:21–23. 10.1038/ng1226 12910270 PMC2864026

[B45] Ochiishi T, Yamauchi T, Terashima T (1998) Regional differences between the immunohistochemical distribution of ca2+/calmodulin-dependent protein kinase ii α and β isoforms in the brainstem of the rat. Brain Res 790:129–140. 10.1016/S0006-8993(98)00058-4 9593859

[B46] Oertel D, Young ED (2004) What’s a cerebellar circuit doing in the auditory system? Trends Neurosci 27:104–110. 10.1016/j.tins.2003.12.001 15102490

[B47] Oertel D, Wright S, Cao X-J, Ferragamo M, Bal R (2011) The multiple functions of t stellate/multipolar/chopper cells in the ventral cochlear nucleus. Hear Res 276:61–69. 10.1016/j.heares.2010.10.018 21056098 PMC3078527

[B48] Owen SF, Liu MH, Kreitzer AC (2019) Thermal constraints on in vivo optogenetic manipulations. Nat Neurosci 22:1061–1065. 10.1038/s41593-019-0422-3 31209378 PMC6592769

[B49] Palmer AR, Shackleton TM, Sumner CJ, Zobay O, Rees A (2013) Classification of frequency response areas in the inferior colliculus reveals continua not discrete classes. J Physiol 591:4003–4025. 10.1113/jphysiol.2013.255943 23753527 PMC3764642

[B50] Parham K, Bonaiuto G, Carlson S, Turner JG, D'Angelo WR, Bross LS, Fox A, Willott JF, Kim DO (2000) Purkinje cell degeneration and control mice: responses of single units in the dorsal cochlear nucleus and the acoustic startle response. Hear Res 148:137–152. 10.1016/S0378-5955(00)00147-7 10978831

[B51] Parras GG, Nieto-Diego J, Carbajal GV, Valdés-Baizabal C, Escera C, Malmierca MS (2017) Neurons along the auditory pathway exhibit a hierarchical organization of prediction error. Nat Commun 8:2148. 10.1038/s41467-017-02038-6 29247159 PMC5732270

[B52] Perry S, Gezelius H, Larhammar M, Hilscher MM, Lamotte d'Incamps B, Leao KE, Kullander K (2015) Firing properties of renshaw cells defined by chrna2 are modulated by hyperpolarizing and small conductance ion currents ih and isk. Eur J Neurosci 41:889–900. 10.1111/ejn.12852 25712471

[B53] Roberts MT, Trussell LO (2010) Molecular layer inhibitory interneurons provide feedforward and lateral inhibition in the dorsal cochlear nucleus. J Neurophysiol 104:2462–2473. 10.1152/jn.00312.2010 20719922 PMC2997026

[B54] Robertson D, Mulders WH (2018) Cholinergic responses of acoustically-characterized cochlear nucleus neurons: an in vivo iontophoretic study in guinea pig. Hear Res 367:97–105. 10.1016/j.heares.2018.07.010 30081246

[B55] Rossant C, Kadir SN, Goodman DFM, Schulman J, Hunter MLD, Saleem AB, Grosmark A, Belluscio M, Denfield GH, Ecker AS, Tolias AS, Solomon S, Buzsaki G, Carandini M, Harris KD (2016) Spike sorting for large, dense electrode arrays. Nat Neurosci 19:634–641. 10.1038/nn.4268 26974951 PMC4817237

[B56] Schneider CA, Rasband WS, Eliceiri KW (2012) NIH image to ImageJ: 25 years of image analysis. Nat Methods 9:671–675. 10.1038/nmeth.2089 22930834 PMC5554542

[B57] Shimano T, Fyk-Kolodziej B, Mirza N, Asako M, Tomoda K, Bledsoe S, Pan ZH, Molitor S, Holt AG (2013) Assessment of the AAV-mediated expression of channelrhodopsin-2 and halorhodopsin in brainstem neurons mediating auditory signaling. Brain Res 1511:138–152. 10.1016/j.brainres.2012.10.030 23088961 PMC4581596

[B58] Shore SE, Koehler S, Oldakowski M, Hughes LF, Syed S (2008) Dorsal cochlear nucleus responses to somatosensory stimulation are enhanced after noise-induced hearing loss. Eur J Neurosci 27:155–168. 10.1111/j.1460-9568.2007.05983.x 18184319 PMC2614620

[B59] Shore SE, Roberts LE, Langguth B (2016) Maladaptive plasticity in tinnitus — triggers, mechanisms and treatment. Nat Rev Neurol 12:150–160. 10.1038/nrneurol.2016.12 26868680 PMC4895692

[B60] Siegle JH, López AC, Patel YA, Abramov K, Ohayon S, Voigts J (2017) Open-Ephys: an open-source, plugin-based platform for multichannel electrophysiology. J Neural Eng 14:e045003. 10.1088/1741-2552/aa5eea 28169219

[B61] Siwani S, França ASC, Mikulovic S, Reis A, Hilscher MM, Edwards SJ, Leão RN, Tort ABL, Kullander K (2018) OLMα2 cells bidirectionally modulate learning. Neuron 99:404–412.e3. 10.1016/j.neuron.2018.06.022 29983324

[B62] Tzounopoulos T (2008) Mechanisms of synaptic plasticity in the dorsal cochlear nucleus: plasticity-induced changes that could underlie tinnitus. Am J Audiol 17:S170–S175. 10.1044/1059-0889(2008/07-0030) 18978197 PMC2804917

[B63] Van Der Walt S, Colbert SC, Varoquaux G (2011) The NumPy array: a structure for efficient numerical computation. Comput Sci Eng 13:22–30. 10.1109/MCSE.2011.37

[B64] Wang X, Zhang C, Szábo G, Sun Q-Q (2013) Distribution of CaMKIIα expression in the brain in vivo, studied by CaMKIIα-GFP mice. Brain Res 1518:9–25. 10.1016/j.brainres.2013.04.042 23632380 PMC3747672

[B65] Willott JF, Erway LC (1998) Genetics of age-related hearing loss in mice. IV. Cochlear pathology and hearing loss in 25 BXD recombinant inbred mouse strains. Hear Res 119:27–36. 10.1016/S0378-5955(98)00029-X 9641316

[B66] Winer JA, Prieto JJ (2001) Layer v in cat primary auditory cortex (ai): cellular architecture and identification of projection neurons. J Comp Neurol 434:379–412. 10.1002/cne.1183 11343289

[B67] Xie R, Manis PB (2014) GABAergic and glycinergic inhibitory synaptic transmission in the ventral cochlear nucleus studied in VGAT channelrhodopsin-2 mice. Front Neural Circuits 8:84. 10.3389/fncir.2014.00084 25104925 PMC4109614

